# Effects of a Web-Based Patient Activation Intervention to Overcome Clinical Inertia on Blood Pressure Control: Cluster Randomized Controlled Trial

**DOI:** 10.2196/jmir.2298

**Published:** 2013-09-04

**Authors:** Jeffrey Thiboutot, Christopher N Sciamanna, Bonita Falkner, Donna K Kephart, Heather L Stuckey, Alan M Adelman, William J Curry, Erik B Lehman

**Affiliations:** ^1^Mount Sinai School of MedicineNew York, NYUnited States; ^2^Pennsylvania State University College of MedicineMS Hershey Medical CenterHershey, PAUnited States; ^3^Jefferson Medical CollegeThomas Jefferson UniversityPhiladelphia, PAUnited States

**Keywords:** hypertension, Internet, tailored-feedback, Web-based

## Abstract

**Background:**

Only approximately half of patients with hypertension have their blood pressure controlled, due in large part to the tendency of primary care providers (PCPs) not to intensify treatment when blood pressure values are elevated.

**Objective:**

This study tested the effect of an intervention designed to help patients ask questions at the point of care to encourage PCPs to appropriately intensify blood pressure treatment.

**Methods:**

PCPs and their patients with hypertension (N=500) were recruited by letter and randomized into 2 study groups: (1) intervention condition in which patients used a fully automated website each month to receive tailored messages suggesting questions to ask their PCP to improve blood pressure control, and (2) control condition in which a similar tool suggested questions to ask about preventive services (eg, cancer screening). The Web-based tool was designed to be used during each of the 12 study months and before scheduled visits with PCPs. The primary outcome was the percentage of patients in both conditions with controlled blood pressure.

**Results:**

Of 500 enrolled patients (intervention condition: n=282; control condition: n=218), 418 (83.6%) completed the 12-month follow-up visit. At baseline, 289 (61.5%) of participants had controlled blood pressure. Most (411/500, 82.2%) participants used the intervention during at least 6 of 12 months and 222 (62.5%) reported asking questions directly from the Web-based tool. There were no group differences in asking about medication intensification and there were no differences in blood pressure control after 12 months between the intervention condition (201/282, 71.3%) and control condition (143/218, 65.6%; *P*=.27) groups. More intervention condition participants discussed having a creatinine test (92, 52.6% vs 49, 35.5%; *P*=.02) and urine protein test (81, 44.8% vs 21, 14.6%; *P*<.001), but no group differences were observed in the rate of testing. The control condition participants reported more frequent discussions about tetanus and pneumonia vaccines and reported more tetanus (30, 13.8% vs 15, 5.3%; *P*=.02) and pneumonia (25, 11.5% vs 16, 5.7%; *P*=.02) vaccinations after 12 months.

**Conclusions:**

The use of an interactive website designed to overcome clinical inertia for hypertension care did not lead to improvements in blood pressure control. Participant adherence to the intervention was high. The control intervention led to positive changes in the use of preventive services (eg, tetanus immunization) and the intervention condition led to more discussions of hypertension-relevant tests (eg, serum creatinine and urine protein). By providing patients with individually tailored questions to ask during PCP visits, this study demonstrated that participants were likely to discuss the questions with PCPs. These discussions did not, however, lead to improvements in blood pressure control.

**Trial Registration:**

ClinicalTrials.gov NCT00377208; http://clinicaltrials.gov/ct2/show/NCT00377208 (Archived by WebCite at http://www.webcitation.org/6IqWiPLon).

## Introduction

Hypertension is one of the most common chronic illnesses in the United States, affecting more than 1 in 4 adults [1]. Hypertension is strongly associated with increased risk of cardiovascular disease, which is estimated to have caused 599,413 deaths in the United States in 2009, or 24.6% of all deaths [2]. Clinical trials have shown that blood pressure control reduces the risk of stroke, myocardial infarction, and heart failure [3]. Despite the known benefits of blood pressure control, in 2008, the National Health and Nutrition Examination Survey (NHANES) observed that only approximately 50% of those diagnosed with hypertension had their blood pressure controlled [1].

Given the contribution of hypertension to cardiovascular disease and the relatively high rates of uncontrolled blood pressure, many intervention methods have been developed and tested. Team-based care, for example, in which the patient’s primary care provider works with other professionals, such as nurses, pharmacists, dietitians, social workers, and community health workers, has consistently been observed to improve blood pressure control [4,5]. The Guide to Community Preventive Services from the Centers for Disease Control and Prevention (CDC) recently concluded that team-based care increased the percentage of patients with controlled blood pressure by 12.0% (interquartile range [IQR] 3.0-19.5, 31 studies) [4]. Despite these recommendations, as fee-for-service care remains the dominant reimbursement model for health care in the United States, disseminating team-based care is challenging without understanding how it could be paid for [6,7].

Engaging patients in their own care, known as *patient activation*, has been increasingly described as a strategy to improve self-management of chronic diseases such as hypertension [8]. One important way for patients to be involved in their care is to ask questions during physician visits. Kravitz and colleagues [9] observed that standardized patients who were instructed to ask specific questions to receive a treatment of depression were more than twice as likely to receive a prescription for an antidepressant medication as those who were instructed to make no request. This is consistent with many studies that report that giving patients reminders to ask providers about tests and treatments they are due to receive, such as vaccines and cancer screenings, increases the likelihood that they receive the recommended care [10,11]. In a meta-analysis, for example, Stone and colleagues [12] observed that giving reminders to patients was consistently effective at increasing adherence with cancer screening guidelines and was more effective than patient education.

We undertook this study to understand whether the same approach could be used with a chronic medical condition such as hypertension. We hypothesized that if patients whose blood pressure was not controlled were reminded to ask specific questions that may lead their provider to intensify their care, that the reminders would increase blood pressure control. The target, therefore, was clinical inertia, or the tendency of providers not to make a change to the plan of care for participants who are not at their treatment target [13-15]. Berlowitz and colleagues [15] observed that patients whose blood pressure was greater than 155/90 mm Hg and whose blood pressure was elevated at a previous visit where the provider made no change, had an intensification made to their blood pressure medications in only 25.6% of visits. Therefore, we hypothesized that the intervention would increase medication intensification among patients whose blood pressure was not at target, which would thereby increase the percentage of patients who achieved standard blood pressure goals. The study was designed as a cluster-randomized trial, common to clinical trials of interventions that are implemented at the level of a larger unit, such as a hospital [16], physician [17], or physician practice [5].

The overall intent of the intervention was to encourage users whose blood pressure was not at goal to ask questions that would lead to medication intensification. We chose to target patients with hypertension that had a history of not being controlled, but did not require all patients at baseline to have uncontrolled hypertension for the following reasons. First, the intervention was designed for a managed care organization (MCO) to make available to the individual patients covered by the MCO. However, MCOs, other than staff-model MCO’s (eg, Kaiser Permanente), are typically unaware of which patients have controlled and uncontrolled blood pressure because they lack access to data from the electronic health record. For that reason, we anticipated that MCOs would make such a tool available to patients without regard to blood pressure values, given findings from Egan and others [1] that blood pressure control in the United States is suboptimal. Second, a number of the recommendations from blood pressure guidelines [18] are for regular tests to be done (eg, kidney function) that are not specific to whether or not blood pressure is controlled. Third, blood pressure control varies over time, so that many patients whose blood pressure is controlled at 1 point in time will have uncontrolled blood pressure at a subsequent visit, requiring additional medications.

## Methods

### Overview

A complete description of the study design and baseline characteristics of participants is published elsewhere [19]. The protocol and all consent forms were approved by the Institutional Review Board of the Pennsylvania State University College of Medicine. This was a randomized controlled trial (NCT00377208).

### Design

#### Randomization

Physicians were randomized to the intervention condition or control condition and, consistent with a cluster-randomized trial design, all patients recruited from a physician were then assigned to the same condition as their physician. Therefore, all interventions pertained to the cluster to which the physician was assigned. For example, for providers assigned to the intervention condition, all of their patients who were enrolled in this study were assigned to the hypertension intervention. To reduce the chances that staff would treat patients differently, particularly while assessing outcomes, staff were blinded to the condition of the provider. Providers and their patients were randomized to 1 of 2 following conditions.

#### Intervention Condition

Participants were instructed to answer questions online once each month and before any visits with their hypertension care provider. Questions focused on the care they had recalled receiving (eg, creatinine testing) and the blood pressure from their most recent doctor visit. Based on their responses and prewritten rules, participants received a brief prewritten tailored feedback message. Each tailored feedback message included a question that the patient should consider asking their provider (eg, “What can you do to help me lower my blood pressure?”) and a lay summary of the guideline recommendation and the evidence underlying the recommendation. All decision rules and tailored messages were based on the Seventh Report of the Joint National Committee on Prevention, Detection, Evaluation, and Treatment of High Blood Pressure (JNC 7) [18] and were reviewed by a nephrologist (BF).

#### Control Condition

Participants randomized to this group received Web-based tailored feedback and were prompted to ask questions during primary care provider (PCP) visits regarding preventive services that they were due to receive. All decision rules and tailored messages were based on guidelines from the United States Preventive Services Task Force (USPSTF; eg, tetanus vaccination, screening for colon cancer). The frequency of activities in the control condition was identical to those in the intervention condition.

### Measures

The primary outcome measure was blood pressure control. Blood pressure was measured by a standardized protocol [20]. As blood pressure control targets are different for those with diabetes and chronic kidney disease (≤130/≤80 mm Hg) than those without these conditions (≤140/≤90 mm Hg), chart reviews were used to determine the prevalence of each [18]. The hypothesized mediating variable was the change in the number of blood pressure medications, which the intervention was intended to increase. Secondary outcomes included changes in the number of hypertension screening tests (eg, urine protein, serum creatinine), also recommended in JNC 7, as well as changes in doctor-patient communication.

Changes in medications and hypertension-related tests (eg, creatinine) were measured by chart abstraction at 12 months after the baseline study visit. The use of preventive services (eg, tetanus vaccination) was measured via patient self-report. The impact of the intervention on doctor-patient communication was measured by a self-reported survey, completed within 72 hours after the participants’ first visits with their hypertension care provider. This exit survey was designed to measure what was discussed during the visit, and provide insight into how the tailored feedback was being used. Similar methods have been studied by 1 of the investigators (CNS) and observed to be accurate for identifying activities that occur during provider visits [21]. All outcome measures were performed similarly for participants in both clusters. For example, participants in the control condition, which focused on preventive services, had their blood pressure measured and their chart reviewed at the same time (eg, 12 months after the baseline study visit) and in the same manner as participants in the intervention condition, which focused on hypertension.

### Recruitment and Study Flow

**Figure 1:**
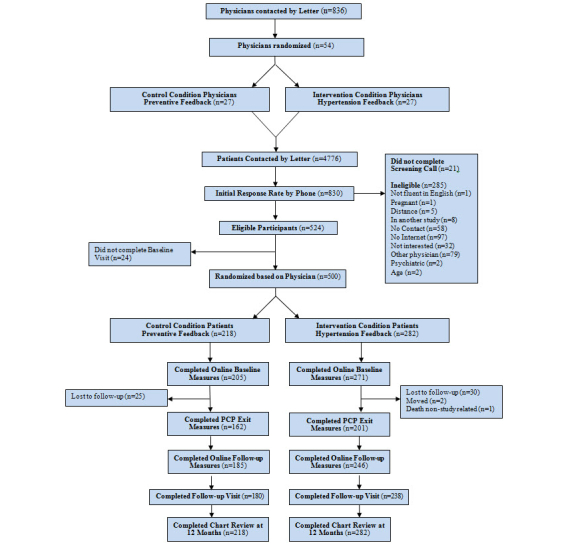
CONSORT diagram of participant flow.

See Figure 1 for the Consolidated Standards of Reporting Trials (CONSORT) diagram of participant flow. Consistent with the cluster-randomized trial design, we first recruited PCPs and then recruited patients to the same condition as their PCP. The PCPs whose practices were located within 40 miles of Penn State Hershey Medical Center (PSHMC) in Central Pennsylvania were recruited. To limit the study to providers engaged primarily in clinical care, recruitment was limited to PCPs who were physicians, board certified in internal medicine or family practice, and who practiced at least 4 half-days each week. To maximize recruitment of minority patients, online census data were used to create a list of zip codes within 40 miles of PSHMC with the highest racial and ethnic minority populations. A list of PCPs within these zip codes was then purchased from a marketing firm (SK&A, A Cegedim Company, Irvine, CA, USA). Recruitment letters were mailed to providers. To minimize the potential for unblinding physicians, which can lead physicians to intervene in other ways, known as *co-intervention* [22], all recruitment letters and discussions with physicians stated that the overall goal of the study was to improve primary and secondary prevention for patients with hypertension. The rationale for this was based on findings by Fontana and colleagues [23], who observed that patients with chronic medical conditions, such as hypertension, are less likely to receive preventive services such as mammography. Study staff members made follow-up phone calls to assess the level of interest of the physicians in having their practice participate in the study. Project staff visited physicians who expressed interest to explain the study more fully and to recruit them into the study.

After getting consent from the PCP, study staff visited the practice to review the charts of patients to identify eligible patients who met the blood pressure and age criteria (Table 1). Patients meeting these criteria were mailed recruitment letters cosigned by their PCP and the study investigator (see Multimedia Appendix 1). Patients interested in participating were then encouraged to call the toll-free study number. During a screening phone call, the study was explained to the patient and the patient was assessed for the remaining inclusion and exclusion criteria (Table 1).

Eligible participants were scheduled for a baseline visit at their physician’s office, where study staff received their consent (see Multimedia Appendix 2), measured their blood pressure, and recorded their current medications. After the baseline visit, participants were considered enrolled and were assigned to the same condition as their PCP. After leaving the baseline visit, participants completed the baseline measures (eg, demographics) on the study website using their personal work or home computer. During the study, scheduled dates of all visits with their PCP were tracked via a question in each monthly survey. After the first visit with their PCP, participants completed an exit survey online to assess what care was provided during the first visit with the PCP (eg, topics discussed, medication changes made; see Multimedia Appendix 3). At the end of the 12-month study, participants completed follow-up self-reported measures on the study website (see Multimedia Appendix 4). At 12 months, participants met for 1 final time with a study staff member in the office of their PCP to measure their blood pressure and record their current medications. There was no cost associated with using the study website.

**Table 1:** Patient inclusion and exclusion criteria.

Criteria	Description
Inclusion	Age ≥21 years
	Fluent in English
	At least 2 high blood pressure readings in the previous 12 months (≥130/≥80 mmHg for patients with diabetes or chronic kidney disease, ≥140/≥90 mmHg without)
	Primary care provider was participating in the study
Exclusion	Receiving care from another physician for hypertension treatment (eg cardiologist)
	Hospitalized for a psychiatric disorder in the past 3 years
	Participating in another clinical research study
	Pregnant or planned to become pregnant in the next 12 months
	Planning on moving out of the area in the next 12 months
	No personal access to the Internet at home or at work
	No personal email account

### Randomization

Randomization was done at the level of the PCP. PCPs were enrolled and randomized into 1 of 2 conditions by selecting an envelope containing a document noting the assigned condition (intervention condition or control condition) from a stack of sealed envelopes, the order of which was generated by the study statistician (EL). All patient participants were assigned to the same condition as their PCP, consistent with a cluster-designed randomized trial [5,16,17]. This was done to eliminate contamination because the intervention had the potential to change the care that the PCP may provide to other patients in the practice. To ensure fidelity to the use of the intervention, participants in both conditions were eligible to receive US $5 for each month they used the website, for a potential total of US $60.

### Intervention Condition

**Figure 2:**
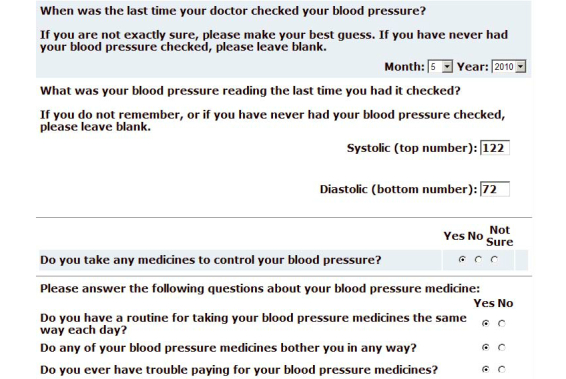
Screenshot of intervention condition monthly survey.

The intervention condition participants received access to the hypertension module of the Web-based intervention for 12 months, which included: (1) Web-based hypertension feedback based on the individual patient’s self-report of health variables (see Figure 2 and Multimedia Appendix 5), decision rules, and tailored feedback based on recommendations from JNC 7 [18], (2) a “pocket chart” that patients could print and take to their doctor visits to help them record their blood pressure that could later be entered into the website, and (3) automated reminders that tracked the dates of upcoming visits with their PCP to remind patients to use the website before physician visits. Participants were expected to use the website at least once each month and received reminder emails if 30 days had elapsed since the last time they used the website.

**Figure 3:**
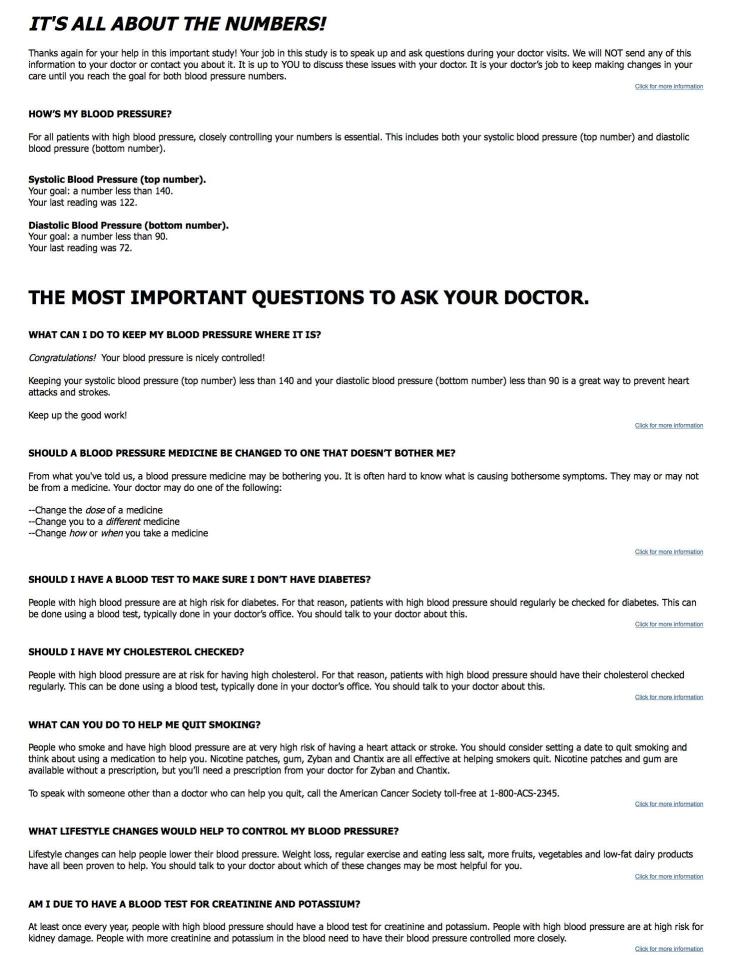
Screenshot of intervention condition feedback from monthly survey.

On the website, patients entered blood pressure values measured at clinical visits and answered questions about their hypertension-related care (eg, date and value of last creatinine blood test). The patient was then provided with onscreen tailored feedback, based on preprogrammed rules adapted from recommendations in JNC 7 [18]. For any situation in which the patient appeared to be in need of a change to their care (eg, blood pressure was higher than JNC 7 goals), the tailored feedback also included a question that the participant should consider asking at the next visit (eg, “Can I lower my blood pressure by drinking less alcohol?”). Participants also received a layperson description of the scientific rationale for the statement, and a link to an external website to provide supporting information for each recommendation (eg, American Heart Association). The feedback was ordered so that the highest priority recommendations appeared closest to the top of the page (see Figure 3 and Multimedia Appendix 6).

The Web-based feedback was based on the hypertension guidelines in JNC 7 [18], which was reviewed for the presence of specific recommendations on hypertension management. Recommendations were ranked based on the strength of the supporting evidence as well as the likelihood of impact from increasing adherence to the recommendation. These recommendations were reviewed by the study’s clinical hypertension expert (BF). For example, although JNC 7 recommends checking potassium before initiating medication therapy, its impact on blood pressure control or on the morbidity from hypertension is likely to be limited compared to the impact of adding a medication to lower the blood pressure value [18]. Given the time constraints of PCP visits [24], it was assumed that patients would be able to ask no more than 1 to 2 questions during physician visits and expect that these questions would be appropriately addressed. For this reason, the prioritization of recommendations was done so that the recommendations that were the most widely accepted and most likely to affect blood pressure control appeared closest to the top of the Web page. For example, the tailored messages on the top focused on the target values of systolic and diastolic blood pressure because these are a consistent focus of recommendations from the JNC 7 [18], the National Committee for Quality Assurance (NCQA) [25], and are considered a measure of high quality of care [26].

The intervention was designed to be used before a visit with the physician who provided the patient’s hypertension care. For that reason, it was essential to track the dates of these visits so the patients could be reminded to use the intervention before these visits. It was assumed that the intervention would be significantly less effective if used long before or following an office visit, as the intervention is designed to activate patients to ask specific questions during visits [27]. For that reason, participants in both conditions received monthly email reminders to use the intervention, in large part to track the date of the next hypertension care provider visit so that users could be prompted to use the intervention before these planned visits. Participants in both conditions then received email reminders to use the site starting 10 days before their physician visit and repeated twice if the participant had not used the site before the planned visit. This had a secondary benefit of assuring that the feedback was based on the most recent data (ie recent laboratory or blood pressure values). This approach was effective in a separate study, where more than 90% of patients used the website in the 2 weeks before a physician visit [28].

An important requirement of the intervention was that patients enter data (eg, blood pressure, creatinine values) that they would typically only receive during visits with a health care provider. For that reason, we created a wallet-sized pocket chart to help patients gather this data during office visits. The participant could then later enter these numbers into the website. Participants were encouraged to print the pocket chart and bring it to their doctor visits and ask their physician to record test results, or ask their physician for the test value and record it themselves.

### Control Condition

The control condition was identical to the intervention condition, except that the content of the control condition intervention focused exclusively on preventive services rather than hypertension (see Multimedia Appendix 7). The control condition participants received the same components of the intervention as intervention condition participants (eg, Web-based personalized feedback, pocket chart, email reminders), but the website focused on preventive services that were not related to hypertension care (eg, mammography screening, tetanus immunizations) and were recommended by the USPSTF (see Multimedia Appendix 8). The control condition, being an active treatment control condition, was designed to improve preventive care and not hypertension care. For example, it was designed not to provide feedback about increasing physical activity, which can lower blood pressure. This control condition design, similar to that used by other investigators [29,30], was chosen to limit attrition and control for contact time.

### Effect Size and Statistical Analysis

The expected effect size was based on a meta-analysis by Stone and colleagues [11] that examined the efficacy of reminders given to patients on rates of adult immunization and cancer screening services. Patient reminders were observed to significantly increase immunizations (odds ratio [OR] 2.5), mammography utilization (OR 2.3), cervical cytology screening (OR 1.7), and colon cancer screening (OR 2.8), effect sizes that were consistent with reviews by other investigators [31-33]. Therefore, the current study was powered to detect an effect size that translates to a more conservative relative risk for blood pressure control of 1.5% to 60% in the intervention condition and 40% in the control condition. Given the cluster design and that practice effects tend to induce positive correlation among patient outcomes, we included in our sample size calculations a conservative intrapractice correlation coefficient (intervention condition) of 0.1. Our power calculations indicated that 12 practices per treatment group (24 total) with at least 200 patients per treatment group (400 total) were needed to detect these differences.

The 2 randomized groups were compared on important demographic and other baseline variables to ensure successful randomization. Student *t* tests and Pearson chi-square tests were used, respectively, to examine between-group differences in continuous and categorical variables. This comparison was done to ensure that randomization created equal groups (Table 2).

Data analysis was focused on the primary hypothesis that a higher percentage of participants in the intervention condition condition, compared to control condition participants, would have controlled blood pressure at 12 months, using intent-to-treat principles [34]. Overall rate of blood pressure control was compared between groups using Pearson chi-square test. The effects of the intervention on continuous blood pressure values were then compared using the Student *t* test. Linear mixed effects modeling was used to control for the potential impact of variables that differed between conditions at baseline (number of blood pressure medications and employment status) [35]. Subgroup analyses were performed to understand the impact of the intervention on individuals whose blood pressure was not controlled at baseline. The data were first analyzed limited to those who followed up at 12 months. Although there are many methods to account for missing data at follow-up, we used the Markov chain Monte Carlo (MCMC) method via the multiple imputation procedure statement (PROC MI) in the SAS statistical analysis software system (SAS Institute, Inc, Cary, NC, USA), as has been used in human immunodeficiency virus clinical trials and in other cluster-randomized trials [36,37]. Most importantly, the point estimates of blood pressure with or without the multiple imputation differed by <1.0%, with neither method yielding results that were near clinical or statistical significance. Because the results were not qualitatively different between these methods, the results are presented using imputed values for all 500 participants randomized at baseline.

**Table 2:** Baseline data comparing characteristics in different conditions.

Characteristic		Total (N=500)	Intervention (n=282)	Control (n=218)	*P* value^a^
Age (years), mean (SD)		60.5 (11.9)	59.6 (12.1)	61.6 (11.4)	.07
Gender (female), n (%)		288 (57.6)	165 (58.5)	123 (56.4)	.64
**Race/ethnicity, n (%)**					
	Non-Hispanic white	375 (75.0)	123 (75.5)	162 (74.3)	.75
	Hispanic	13 (2.6)	10 (3.5)	3 (1.4)	.13
**Background, n (%)**					
	Education (college ≥4 years)	202 (42.4)	113 (41.7)	89 (43.4)	.71
	Income (≤US $50,000)	221 (49.3)	124 (49.0)	97 (49.7)	.88
	Employed for wages	214 (45.0)	140 (51.7)	74 (36.1)	<.001
**Health**					
	Body mass index, mean (SD)	32.4 (7.4)	32.1 (7.3)	32.7 (7.6)	.42
	Smoking, n (%)	41 (8.6)	21 (7.8)	20 (9.8)	.44
	Diabetes, n (%)	104 (22.0)	61 (22.6)	43 (21.3)	.73
	Health (very good/excellent), n (%)	160 (33.6)	88 (32.5)	72 (35.1)	.54
**Blood pressure (BP)**					
	Systolic (mm Hg), mean (SD)	132.6 (15.0)	132.7 (14.9)	132.4 (15.2)	.84
	Diastolic (mm Hg), mean (SD)	75.5 (11.0)	75.7 (11.1)	75.2 (10.9)	.62
	Systolic controlled, n (%)	303 (64.5)	181 (67.5)	122 (60.4)	.11
	Diastolic controlled, n (%)	405 (86.2)	203 (85.8)	175 (86.6)	.80
	Overall controlled, n (%)	289 (61.5)	170 (63.4)	119 (58.9)	.32
	Number of BP medications, n (%)	1.0 (1.61)	1.0 (1.51)	1.0 (1.73)	.02
**Internet use, n (%)**					
	Internet use for health, ≥once/month	94 (20.9)	52 (20.4)	42 (21.7)	.75
	Used Internet before a physician visit	227 (51.1)	140 (54.9)	87 (46.0)	.06

^a^ *P* value from 2-sample *t* test for continuous outcomes and Pearson chi-square test for categorical outcomes.

## Results

### Summary

Five university physicians group clinics associated with Hershey Medical Center and 836 family practices were contacted to enroll in our study. Of the physicians contacted, 54 (6.4%) responded and agreed to participate. Consistent with a cluster-randomized design, randomization was at the level of the provider, and each cluster included the provider and all patients of that single provider who were enrolled in the study. Therefore, all patients recruited were assigned to the condition (intervention condition or control condition) that the provider had been randomly assigned, and all analyses were performed at the level of the cluster, in this case the provider. After a medical record chart review, patients of enrolled physicians with a diagnosis of hypertension (n=4776) were sent recruitment letters inviting them to participate in the study. Of those who were sent a letter, 828 (17.3%) responded and 812 (17%) were able to be contacted and screened for eligibility. Eligible participants (n=528) were scheduled for a baseline visit at which 3 consecutive blood pressures were measured. Of those scheduled, 500 completed the baseline visit and 218 participants were enrolled into the control condition (prevention) and 282 into the intervention condition (hypertension). Following the baseline visit, 476 (95.2%) participants logged onto the website and completed the online baseline measures. From the 476 participants who completed the baseline measures questionnaire, demographic data as well as baseline secondary outcome data were collected (Table 2). Following their first visit with their PCP, 363 (72.6%) participants completed a survey to record what occurred during the visit. After 12 months, 418 (83.6%) returned for their follow-up visit.

### Fidelity

**Figure 4:**
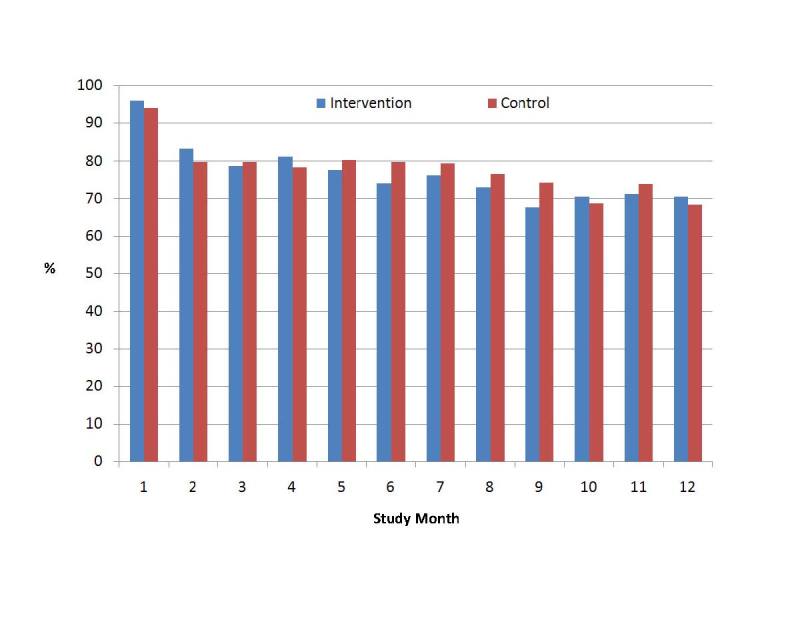
Percentage of participants using the intervention during each of the 12 study months.

**Figure 5:**
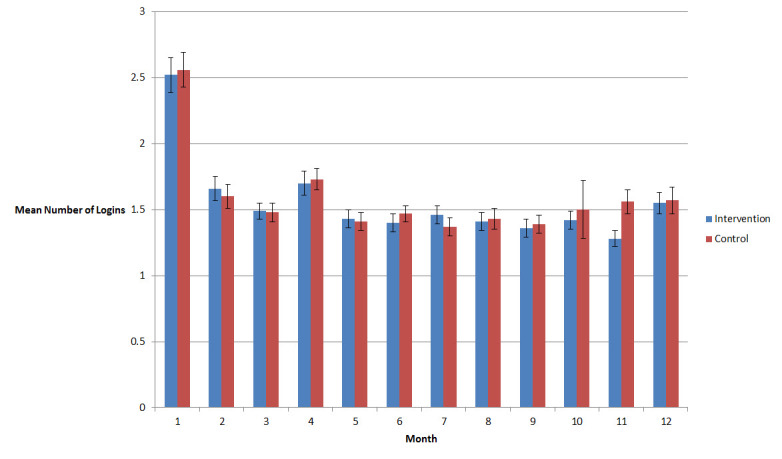
Mean (± standard error) number of log-ins per month in both conditions in each of the 12 study months.

As stated previously, the intervention was designed to be used by answering a series of questions and reviewing tailored feedback at least once each month. Of the 500 participants, 411 (82.2%) used the intervention during at least 6 of 12 months, and 174 (34.8%) logged into and used the website each of the 12 months enrolled in the study (Figure 4). Adherence was monitored electronically by number of months in which participants logged in (Figure 5). There was no significant difference in use of the intervention observed between study groups.

### Baseline Characteristics

Table 2 reports the baseline characteristics. There were no significant differences in most variables between study groups, including the demographic variables of age, gender, race, ethnicity, education, and income. Mean systolic blood pressure, for example, was 132.7 mm Hg among intervention condition participants and 132.4 mm Hg among control condition participants (*P*=.84). Similarly, the percentage of participants whose blood pressure was controlled at baseline was similar between groups (170/268, 63.43% in intervention condition; 119/202, 58.9% in control condition; *P*=.32). At baseline, the rates of blood pressure control did not differ between intervention condition and control condition participants or between those enrolled at university-based primary care practices versus community-based primary care practices (data not shown). However, there were significant group differences in 2 variables. More intervention condition participants were employed for wages (140/271, 51.7% vs 74/205, 36.1%; *P*<.001) and control condition participants used a greater number of blood pressure medications (1.73 vs 1.51; *P*=.02) than intervention condition participants. It was also observed that only 1.0% of participants enrolled in the study were uninsured; this is much lower than the Behavioral Risk Factor Surveillance Survey (BRFSS) results from 2007 to 2008 of 15.4% [38].

### Primary Outcomes

Table 3 reports blood pressure outcomes at 12 months. The overall rate of participants with controlled blood pressure increased from 312 of 500 (62.4%) at baseline to 344 of 500 (68.8%) at 12 months. No significant difference was observed between study groups with respect to rates of blood pressure control (intervention condition: 201/282, 71.3%; control condition: 143/218 control, 65.6%; *P*=.31). Similar results were observed when blood pressure was examined as a continuous variable and when the results were expressed as continuous changes within groups. For example, the mean systolic blood pressure at 12 months was not significantly different between conditions (intervention condition: mean 128.3, SD 13.5; control condition: mean 128.9, SD 14.4; *P*=.88). Table 3 shows similar results were found for mean systolic and diastolic blood pressures, and systolic and diastolic control rates. Even after adjusting for baseline differences in the number of blood pressure medications and employment status, blood pressure control in the intervention condition condition was no greater than in the control condition condition after 12 months.

Because the goal of the intervention was to intervene on patients with uncontrolled blood pressure, a subgroup analysis was performed that was limited to those participants whose blood pressure was uncontrolled at baseline. Of the 188 participants found to be uncontrolled at baseline, 87 (46.3%) were controlled at 12-month follow-up. However, no significant difference was observed in blood pressure control rates between study groups (intervention condition: 47/103, 45.6%; control condition: 40/85, 47.1%; *P*=.57).

### Secondary Outcomes

Table 4 presents the impact of the intervention on doctor–patient communication based on data from the exit survey after the first PCP visit during the study. This survey was designed to measure what was discussed during the visit to understand how the tailored feedback was used. Most participants (222/355, 62.5%) reported asking a question that was suggested by the tailored feedback during the visit with their PCP. As expected, there were significant differences in the topics discussed during visits by intervention condition and control condition participants. For example, more control condition participants than intervention condition participants discussed having a tetanus vaccine (50/141, 35.5% vs 28/166, 16.9%; *P*=.02) and a pneumonia vaccination (39/135, 28.9 vs 23/160, 14.4%; *P*=.01). Similarly, more intervention condition participants than control condition participants discussed having serum creatinine tested (92/175, 52.6% vs 49/138, 35.5%; *P*=.02) and urine protein tested (81/181, 44.8% vs 21/144, 14.6%; *P*<.001).

Table 5 presents changes in medications and changes in preventive services and hypertension screening tests between conditions. Changes in medications and hypertension-related tests (eg, creatinine) were measured by chart abstraction at 12 months, whereas the use of preventive services (eg, tetanus vaccination) was measured via patient self-report. No significant difference was observed in the change in number of blood pressure medications used in each group over the 12-month study (–0.17 intervention, –0.28 control; *P*=.64). For preventive services, significantly more participants in the control condition reported receiving a tetanus vaccination in the past year (30/218, 13.8 vs 15/282, 5.3%; *P*=.02) and pneumonia vaccination in the past year (25/218, 11.5% vs 16/282, 5.7%; *P*=.02). However, no differences were observed in the percentage of participants in the intervention condition and control condition who reported receiving an influenza vaccine or colonoscopy. Hypertension screening tests during the intervention also did not differ between conditions. For example, based on chart abstraction, 211 of 282 (74.8%) intervention condition participants had their creatinine tested versus 156 of 218 (71.6%) of control condition participants (*P*=.56). Similar results were observed for urine protein and serum potassium testing during the 12 study months.

**Table 3:** Primary blood pressure (BP) outcomes.

Outcome		Total	Intervention	Control	*P* value^a^
**All participants (N=500)**					
	Systolic BP (mm Hg), mean (SD)	128.5 (13.9)	128.3 (13.5)	128.9 (14.4)	.88
	Diastolic BP (mm Hg), mean (SD)	74.1 (9.2)	73.8 (8.9)	74.4 (9.6)	.15
	Systolic BP controlled, n (%)	372 (74.4)	206 (76.6)	156 (71.6)	.35
	Diastolic BP controlled, n (%)	447 (89.4)	254 (90.1)	193 (88.5)	.59
	Overall BP controlled, n (%)	344 (68.8)	201 (71.3)	143 (65.6)	.27
**Participants uncontrolled at baseline (n=188)**					
	Systolic BP (mm Hg), mean (SD)	135.4 (13.5)	134.9 (13.3)	136.1 (13.9)	.83
	Diastolic BP (mm Hg), mean (SD)	77.0 (10.1)	77.3 (9.5)	76.7 (10.8)	.79
	Systolic BP controlled, n (%)	103 (54.8)	58 (56.3)	45 (52.9)	.89
	Diastolic BP controlled, n (%)	152 (80.9)	81 (78.6)	71 (83.5)	.51
	Overall BP control, n (%)	87 (46.3)	47 (45.6)	40 (47.1)	.57

^a^ *P* value from 2-sample *t* test for continuous outcomes and Pearson chi-square test for categorical outcomes.

**Table 4:** Impact on doctor–patient communication.

Self-reported outcomes		Total n (%)	Intervention condition n (%)	Control condition n (%)	*P* value^a^
**General**					
	Asked any questions from the website	222 (62.5)	125 (63.8)	97 (61.0)	.52
	Discussed notes about website at visit	143 (42.3)	76 (39.8)	70 (45.5)	.37
**Control**					
	Discussed having a tetanus shot	78 (25.4)	28 (16.9)	50 (35.5)	<.001
	Discussed having a pneumonia shot	62 (21.0)	23 (14.4)	39 (28.9)	.01
	Discussed having a flu shot	143 (45.7)	74 (43.3)	69 (48.6)	.94
	Discussed having a test for colon cancer	76 (24.9)	38 (22.8)	38 (27.5)	.55
**Intervention**					
	Discussed what your last blood pressure numbers were	309 (87.3)	171 (87.2)	138 (87.3)	.97
	Discussed having creatinine tested	141 (45.1)	92 (52.6)	49 (35.5)	.02
	Discussed urine test for protein	102 (31.4)	81 (44.8)	21 (14.6)	<.001
	Discussed secondary causes of hypertension	20 (7.2)	10 (6.7)	10 (7.8)	.52
	Discussed changing to blood pressure medication that works better for you	27 (9.3)	17 (10.7)	10 (7.6)	.47
	Discussed more frequent visits until blood pressure controlled	38 (13.7)	24 (16.2)	14 (10.9)	.42
	Doctor recommended starting a new blood pressure medication	34 (10.3)	21 (11.7)	13 (8.7)	.62
	Doctor recommended increasing dose of a blood pressure medication	31 (9.9)	18 (10.7)	13 (9.0)	.52

^a^ *P* value from Pearson chi-square test for categorical outcomes.

**Table 5:** Secondary outcomes of changes in medications and preventive and hypertension screening tests.

Secondary outcomes		Total (n=500)	Intervention (n=282)	Control (n=218)	*P* value^a^
**Medications, mean (SD)**					
	Total number of medications at baseline	1.61 (1.0)	1.51 (1.0)	1.73 (1.0)	.16
	Total number of medications at follow-up	1.39 (1.1)	1.34 (1.1)	1.45 (1.1)	.64
	Change in number of medications	–0.22 (0.93)	–0.17 (0.92)	-0.28 (0.93)	.64
**Preventive services, n (%)**					
	Tetanus vaccine within 1 year	45 (9.0)	15 (5.3)	30 (13.8)	.02
	Pneumonia vaccine within 1 year	41 (8.2)	16 (5.7)	25 (11.5)	.02
	Influenza vaccine within 1 year	280 (56.0)	152 (53.9)	128 (58.7)	.81
	Colonoscopy within 1 year	37 (7.4)	22 (7.8)	15 (6.9)	.72
**Hypertension screening tests, n (%)**					
	Serum creatinine tested within 1 year	367 (73.4)	211 (74.8)	156 (71.6)	.56
	Urine protein tested within 1 year	144 (28.8)	86 (30.5)	58 (26.6)	.26
	Serum potassium tested within 1 year	362 (72.4)	209 (74.1)	153 (70.2)	.31

^a^ *P* value from 2-sample *t* test for continuous outcomes and Pearson chi-square test for categorical outcomes.

## Discussion

### Principal Findings

The present study evaluated the efficacy of an intervention designed to prompt patients to ask questions about their blood pressure control. We hypothesized that by encouraging patients to ask questions with a focus on questions aimed at improving control (“What can you help me do to lower my blood pressure?”), physicians would give patients higher doses of blood pressure medications or additional medications, which would improve blood pressure control more in the intervention condition. The results indicated, however, that the intervention did not improve blood pressure control. Although the intervention led to more discussions of some hypertension-related screening tests (eg, creatinine testing), it did not lead to improvements in blood pressure control. At baseline, 312 of 500 (62.4%) participants had their systolic and diastolic blood pressure controlled as per JNC 7 guidelines [18]. After 12 months of the intervention, those rates were higher, but not significantly different (intervention condition: 201/282, 71.3%; control condition: 143/218, 65.6%; *P*=.27). Similarly, blood pressure improvements were not observed when blood pressure was expressed as a continuous variable or when the analysis was limited to the subgroup of individuals whose blood pressure was uncontrolled at baseline (n=188). However, participants in the control condition group who were prompted to ask questions about preventive services, were more likely to receive a tetanus vaccine (30/218, 13.8% vs 15/282, 5.3%; *P*=.02) and a pneumonia vaccine (25/218, 11.5% vs 16/282, 5.7%; *P*=.02). These findings were consistent with previous studies observing that patient reminders improve preventive service utilization [11].

There are several possible hypotheses to explain why no effect on blood pressure was observed. First, patients may not have been comfortable asking for intensifications to their medication treatment plan because of a concern about questioning the expertise of the provider. Although Kravitz and colleagues [9] observed that prompting standardized patients to ask for a depression treatment increased the chances of receiving treatment, we believe that asking for a drug intensification for hypertension is a very different act. In a clinical encounter for depression, for example, the patient possesses more data than the provider upon which decisions will be made (eg, depressive symptoms). In a clinical encounter for hypertension, this is reversed; the provider typically has more data than the patient (eg, blood pressure values). Because of this difference, encouraging patients to ask questions about their blood pressure control has inherent limitations, making this intervention strategy questionable for this setting. The patient may or may not be aware of their blood pressure at the time of the visit. The blood pressure in the United States is typically measured by a nurse and not a physician, and the nurse may or may not tell the patient the value of their blood pressure. The intervention, however, was designed to prompt patients to become aware of their blood pressure by specifically asking for it. For that reason, two-thirds of participants in the intervention condition entered a blood pressure value. For the other third, however, their lack of awareness of their blood pressure suggests that this method may be of limited use for blood pressure control, unless home blood pressure monitoring is used. Simply put, without knowing the blood pressure, patients would have nothing to ask about. We are currently studying the impact of a similar intervention for asthma (RO1HL088590) which will be more similar to the depression study by Kravitz and colleagues [9] because asthma care is also based on symptoms.

Asking for medication intensification may have been perceived by patients as questioning the judgment of the provider, which may have created a barrier to asking for medication intensifications. This is consistent with the observation, in Table 4, that the intervention led to more conversations about testing for creatinine and urine protein, but no differences in conversations about intensifying medications. It is possible that, if home monitoring had been used, patients may have been more likely to ask for medication intensifications. This situation would be more akin to depression in which the patient has all of the data, which doctors typically do not ask for. We hypothesize that if a patient entered the visit, as suggested by some investigators [39], with an average of 10 blood pressure values that he/she knew were too high, the results of the study may have been different.

A second possible reason for the lack of effect may be the general lack of awareness of the significance of a blood pressure value that is not at target. Although professionals view a systolic blood pressure of 160 mm Hg very differently from 140 mm Hg, these differences are likely not as meaningful to patients. Wright-Nunes and colleagues [40] observed that, even among patients with chronic kidney disease for whom blood pressure control is critical, only 48% of participants identified the correct blood pressure goal, and those who correctly identified their goal had a mean systolic blood pressure 9.96 mm Hg less than those who could not [40]. If the study had limited participation to those with Stage 2 hypertension (systolic ≥160 mm Hg, diastolic ≥100 mm Hg), for example, both providers and patients may have been more responsive to the interventions, believing that the distance from current control to the goal was further. In addition, the lack of symptoms for hypertension removes a key incentive for asking when it is not controlled. Although patients with symptomatic conditions (eg, asthma, depression, migraine) are prompted to ask for treatment intensifications to feel better, patients with conditions that have few symptoms (eg, hypertension, high cholesterol, type 2 diabetes) lack the symptom trigger to request treatment intensifications.

A third possible reason is that blood pressure varies significantly from measurement to measurement, yet the decision rule that created the tailored message was based on just 1 blood pressure measurement [41]. This may have led providers to be less influenced by a request for intensification or created uncertainty in patients who may have presented a barrier to asking. There are several clinical settings in which an elevated blood pressure may not necessitate a dose adjustment [18]. For example, if a medication dose is increased, it may take up to 4 to 6 weeks to see the full effect on blood pressure, so it would be prudent to wait before making a dose change [18]. Also, if the blood pressure has generally been controlled and the blood pressure is in the mild hypertensive range during 1 visit, it may not be appropriate to intensify treatment at the visit. For this reason, Berlowitz and colleagues [15] defined clinical inertia only in patients whose blood pressure was elevated at a prior visit and at a second visit, with no change being made at the second visit. The treatment algorithm we put in place was not sensitive to trends in the blood pressure over time and whether an elevated blood pressure was a 1-time event, such as a patient is having back pain, which often elevates systolic blood pressure. In those situations, deferring decisions on blood pressure medications to a subsequent visit, using home blood pressure monitoring, or ambulatory blood pressure monitoring would be reasonable clinical decisions. Had the intervention been integrated into an electronic health record (eg, patient portal) to generate trend graphs, or used structured repeated home blood pressure monitoring to better determine the blood pressure trend and average, the results may have been different. However, although Hyman and colleagues [42] encouraged the use of ambulatory blood pressure and electronic pill bottle data to reduce physician uncertainty as a potential barrier to intensification, this additional data did not lead to different differences in blood pressure.

Finally, a fourth possible reason for the lack of effect was the impact of secular trends of blood pressure in the United States. Hypertension control improved significantly between 1988 and 2008, which limited the ability of the study to affect blood pressure control because more patients were controlled than anticipated in our power calculations [1]. Egan and colleagues [1] observed that blood pressure control improved from 27.3% (95% CI 25.6-29.1) in 1988-1994 to 50.1% (95% CI 46.8-53.5; *P*=.006) in 2007-2008. Although the level of control was higher than expected, it is noteworthy that the intervention had no impact on the sizeable minority of patients whose blood pressure was uncontrolled at baseline.

### Limitations

This study does have some limitations. First, the patients may not have used the intervention before doctor visits or asked questions during doctor visits and the study did not collect the data to assure that these were done. However, mean use per month (± standard error; as seen in Figure 5) met the goals for the study (≥1/month) and was similar between conditions. We did not have access to time use data, which may have differed between conditions. Because the only activity that participants in each condition were able to do on the site was to read approximately 1 page of tailored text feedback, it is unlikely that this would differ greatly between conditions. Also, each tailored message was accompanied by a link to an external site that provided additional information on the topic (eg, the website for the American Heart Association), and tracking the time spent on those external sites was not technically feasible to our developers at the time this study was conducted. A second design limitation of the study is that we chose not to audio or video record all encounters because of concerns about reactivity. Although Kravitz and colleagues [9] used standardized patients to measure changes in care, the design of the current study was to understand whether typical patients would receive different care as a result of the intervention. Future studies may require audio recording to understand which questions were asked and in what way and more detailed Web tracking to understand which pages were viewed and which links were clicked by participants. In addition, audio recording would be a useful future adjunct to understand the impact of patients proactively asking such questions on what occurs during visits and how doctors treat patients. Recently, for example, Gudzune and colleagues [43] used audio recording to detect that primary care providers demonstrated less emotional rapport with overweight and obese patients, potentially weakening the patient-physician relationship. It is possible that the intervention used in this study may have had a negative impact that would be hard to detect without such audio recording.

Second, a limitation of the study was the management and use of blood pressure values. Patients were encouraged to enter the most recent blood pressure value onto the website, which then generated tailored feedback based, in part, on that number. However, approximately one-third of participants did not enter a blood pressure value because they were unaware of their blood pressure. Even if the participant had entered a blood pressure value, during the subsequent visit to their provider, that value would likely have been different. This situation may have created confusion and uncertainty for patients, undermining their desire to ask for treatment intensifications. This would not be the case, for example, for tetanus vaccination, which is stable over time. To address this limitation, future studies should consider using home blood pressure monitoring, so that patients are prompted to ask questions based on their average home values. Powers and colleagues [41] observed that the mean of 5 home measurements was more accurate for categorizing blood pressure as being high than a single pressure value measured in the office. Green and colleagues [44], for example, encouraged participants to monitor their blood pressure at home, taught them the goals of the numbers, and also how to use an online patient portal (eg, secure email, refilling medications, viewing their health data), yet this did not significantly increase the percentage of patients whose blood pressure was controlled. However, the study by Green and colleagues did not include a patient activation component as in this study, in which patients were encouraged to ask specific questions in response to specific blood pressure values. Future studies may consider examining the impact of combining both elements (blood pressure monitoring plus patient activation questions) on blood pressure control because both may be necessary to assist patients in overcoming clinical inertia in their care.

A third limitation of the study is that the level of blood pressure control observed at baseline in this study was higher than anticipated. The study was powered to detect a 60% blood pressure control rate in the intervention condition versus 40% in the control condition, yet 61.5% of patients had controlled blood pressure at baseline. Enrolling patients whose blood pressure was controlled or nearly controlled lessened the likelihood that patients would see a message from the program that convinced them that their blood pressure was sufficiently far from the goal to talk to their doctor about changing their medications. If the study had limited participation to those with Stage 2 hypertension (systolic ≥160 mm Hg, diastolic ≥100 mm Hg), for example, both providers and patients may have been more responsive to the interventions, believing that the distance from current control to the goal was further. However, if more of the same type of patients were enrolled, it is unlikely that the results would have been different. Of the 188 patients whose blood pressure was not controlled at baseline, blood pressure control at 12 months was slightly higher in the control condition than the intervention condition, suggesting that inflating the sample size would not have changed the outcomes.

Fourth, as in most clinical trials, ours was in a limited geographic region with patient profiles that do not match the target population in the entire United States. Only 1% of participants in this study were uninsured, for example, compared to 4% of primary care patients nationwide [45]. Also, the study included lower rates of African-Americans and Hispanic adults than in the United States’ population. In 2007, according the United States Census Bureau, 15% of adults in the United States were Hispanic or Latino and 63% were non-Hispanic white compared with 2.6% and 75.0% in the present study. Although efforts were made to recruit from communities with higher minority representation, Dauphin County in central Pennsylvania has a markedly lower rate of minority representation than the rest of the nation, making this challenging. We recognize, therefore, that the results may not be generalizable to populations with higher levels of minority representation. In addition, similar to other studies of educational interventions for hypertension [46,47], we excluded patients recently hospitalized for a mental illness because of concerns that this may increase losses to follow-up. However, excluding such individuals is unlikely to affect the external validity of the findings because less than 1% of adults are hospitalized for mental illness each year [48].

A fifth potential limitation is that detailed covariate data were not collected and may have differed between conditions, yet were not adjusted for. Physical activity, alcohol intake, and salt intake each can influence blood pressure [18], yet were not measured. In addition, clinical inertia is greatest when a change was made at the previous visit, yet this was not assessed [15]. Although randomization was used, employment status differed significantly between conditions, so it is possible that other potential confounders differed between conditions as well. We were able to adjust for differences in employment status and number of hypertension medications; thereby, minimizing the impact of differences in these variables on the outcomes, but this would not be possible for unmeasured potential confounders. Given the randomized design, however, the likelihood that these variables differed significantly between conditions is low, given the similarity between conditions on age, gender, race, and other medical conditions (Table 2). For example, body mass index, which is associated with physical activity level [49], was nearly identical between conditions (intervention condition: mean 32.1; control condition mean 32.7; *P*=.42].

### Conclusions

There are several strengths to our study. First, the study used an active treatment control group. Not only did this limit participant attrition and control for contact time, the active treatment control condition provided data to document that the intervention was effective at increasing preventive care, limiting concerns over whether participants had actually used the intervention as it was designed. Second, using the Internet as a communication medium makes what is learned easily disseminated. Although most Web-based studies to date have not shown major health benefits (eg, weight control) [50], some interventions (eg, sleep) have shown benefits [51]. Understanding how best to use this medium, given the low potential per-user cost and wide disseminability, makes studies such as this critical to perform. Although participants in this study were paid and may have checked-in in a perfunctory manner to qualify for the reimbursement because the task being asked was relatively minor (review 1 page of tailored feedback), this would seem unlikely. Also, at the present time, much research is underway to test the impact of gaming elements [52,53] and social elements [54], both of which have the potential to increase engagement without directly compensating participants. Despite low rates of adherence to Web-based interventions [55,56], we observed high rates of fidelity (>70% of all study months) by asking patients to complete a brief Web-based survey, leading to tailored feedback, each month and before provider visits. This intervention structure, monthly use of an online tool, could be used to potentially impact the care of patients experiencing a range of conditions.

## Multimedia Appendix 1

Patient recruitment letter.

## Multimedia Appendix 2

IRB approved informed consent form.

## Multimedia Appendix 3

Patient online exit survey.

## Multimedia Appendix 4

Patient online follow up measures.

## Multimedia Appendix 5

Intervention online monthly survey.

## Multimedia Appendix 6

Intervention online feedback.

## Multimedia Appendix 7

Preventive online monthly survey.

## Multimedia Appendix 8

Preventive online feedback.

## Multimedia Appendix 9

CONSORT-EHEALTH checklist V1.6.2 [57].

## Abbreviations

BRFSS: Behavioral Risk Factor Surveillance Survey
CDC: Centers for Disease Control and Prevention
CONSORT: Consolidated Standards of Reporting Trials
JNC 7: Seventh Report of the Joint National Committee on Prevention, Detection, Evaluation, and Treatment of High Blood Pressure
MCMC: Markov chain Monte Carlo
MCO: managed care organization
NCQA: National Committee for Quality Assurance
NHANES: National Health and Nutrition Examination Survey
OR: odds ratio
PCP: primary care provider
PSHMC: Penn State Hershey Medical Center
USPSTF: United States Preventive Services Task Force
