# Supplementary figures and images for: Effects of a Web-Based Patient Activation Intervention to Overcome Clinical Inertia on Blood Pressure Control: Cluster Randomized Controlled Trial
Source: J Med Internet Res. 2013 Sep 4;15(9):e158. doi: 10.2196/jmir.2298 (PMC3785979; doi:10.2196/jmir.2298)

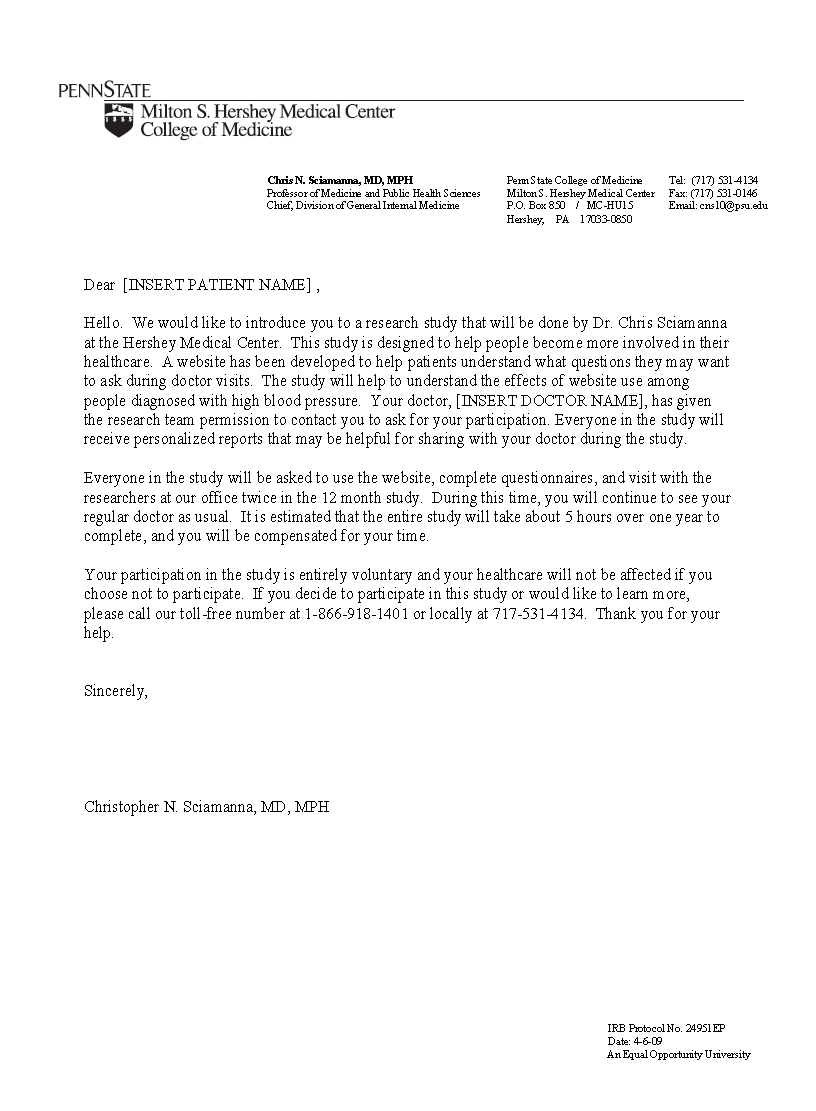

Supplement: Supplementary file 1 [file jmir_v15i9e158_app1.png]
