# Supplementary material for: Effects of a Web-Based Patient Activation Intervention to Overcome Clinical Inertia on Blood Pressure Control: Cluster Randomized Controlled Trial
Source: J Med Internet Res. 2013 Sep 4;15(9):e158. doi: 10.2196/jmir.2298 (PMC3785979; doi:10.2196/jmir.2298)
Supplement: Supplementary file 2 [file jmir_v15i9e158_app2.pdf]

CONSENT FOR RESEARCH
Penn State College of Medicine
The Milton S. Hershey Medical Center

HSPO USE ONLY
DO NOT REMOVE OR MODIFY
This form is not valid unless this box includes an expiration date.

IRB Approval Expires After 05/31/2010 CPW

Penn State College of Medicine
Institutional Review Board

Title of Project: Efficacy of web-based feedback to improve blood pressure control

Principal Investigator: Christopher N. Sciamanna, MD, MPH
Other Investigators:, Michelle Schaffer, PhD, Heather L. Stuckey, D. Ed., Erik B. Lehman M.S., Danielle Loos, BS, Jolene Esposito, BS, Jennifer Poger, BA, Aja Binette, MA, Jeffrey Thiboutot, Taylor Olmsted, Justin Torrente, Chengwu Yang, MD, Ph.D., Linda Kinney, MPA, & Lindsey Brubaker, MPH

Participant's Printed Name: ____________________________

This is a research study. Research studies include only people who want to take part. This form gives you information about this research, which will be discussed with you. It may contain words or procedures that you don't understand. Please ask questions about anything that is unclear to you. Discuss it with your family and friends and take your time to make your decision.

1. Purpose of the Research:

The purpose of this research is to understand the impact of providing individuals with hypertension access to a website that provides automated, tailored feedback. We believe the website will help patients to ask questions that lead to productive patient-physician interactions and improved quality of care and control of hypertension.

Approximately 500 people will take part in this research in Central Pennsylvania.

You are being offered the opportunity to take part in this research because the doctor that you see for your hypertension care is part of the Division of General Internal Medicine at Hershey Medical Center or a Primary Care Provider in the Central Pennsylvania area that chose to participate in our study.

2. Procedures to be Followed:

This study consists of 2 visits, spaced 12 months apart, and website use. If you are interested in the study, you will come to _______ two times over the next 12 months. Your first visit will determine whether you will be eligible for the research study. During this first visit we will review and complete a consent document and will measure your blood pressure and document all of your medications. Once you are considered to be a

good fit for the study, you will be provided the website address and a login and password to complete online surveys. Survey questions may ask you for your last blood pressure numbers, medicines you take, and dates of your last vaccines. You are free to skip any questions that you would prefer not to answer.

You will then be randomly placed in one of two groups (by a method like flipping a coin). One group will login the website and receive information about hypertension care at least once each month for 12 months. The second group will login to the website and receive information about preventive care (such as cholesterol screening and colon cancer screening) at least once a month for the 12 months. Because this is a research study the group that you will be placed in will be determined by chance, so you cannot choose which group you will be in. You need to be willing to participate regardless of which group you are placed in.

You will receive emails prompting you to use the website every 30 days, after your first scheduled doctor's visit, and at the end of the 12 months.

In 12 months, you will complete additional surveys online and then return for your second and final visit. Once again, you are free to skip any questions on the surveys that you would prefer not to answer. At this visit your blood pressure will be measured and we will document all your medications.

Research staff will copy information from your medical record at your doctor's office about hypertension quality of care measures (for example, serum creatinine testing, hypertension specialist consultations). Your medical chart may be reviewed like this for one year before and after you are in the study.

3. <u>Discomforts and Risks:</u>

The main discomforts or risks in this study are loss of confidentiality and any psychological stress induced by the questions in the surveys. These risks are rare.

4. <u>Possible Benefits:</u>

a. Possible benefits to the participant:
The possible benefits you may experience from the website include better communication with your provider and improved care. There is no guarantee that you will benefit from being in this research.

b. Possible benefits to others:
The results of this research may provide a cost-effective way to improve hypertension and preventive care.

5. <u>Other Options that Could be Used Instead of this Research:</u>

You do not have to take part in this research study.

Because it is experimental, the website access offered in this research is only available to you if you take part in the research study.

6. <u>Time Duration of the Procedures and Study:</u>

If you agree to take part in this study your involvement will last approximately 12 months. You will be asked to come to the research office at _________ 2 times. Each visit will take approximately 20 minutes. Your use of the website each month should take about 5 minutes.

7. <u>Statement of Confidentiality:</u>

a. Privacy and confidentiality measures
Your research records that are reviewed, stored, and analyzed at The Milton S. Hershey Medical Center (HMC) and Penn State College of Medicine (PSU) will be kept on a secure, password protected website. Any paper documents will be kept in a locked file draw or password protected computer file. Your research records will be labeled by a code number and will be accessible only to staff on this research study.

In the event of any publication or presentation resulting from the research, no personally identifiable information will be shared.

7b. The use of private health information:

Health information about you will be collected if you choose to be part of this research study. Health information is protected by law as explained in the HMC Privacy Notice. A copy of the HMC Privacy Notice is included in the research folder you will be receiving today. At The Milton S. Hershey Medical Center (HMC) and Penn State College of Medicine (PSU) your information will only be used or shared as explained and authorized in this consent form or when required by law. To participate in this research you must allow your primary care provider, _____________________, to share and the research team to use your health information. If you do not want us to use your protected health information, you may not participate in this research. The research-related website is investigational; therefore, it is not available unless you allow the use of your health information that is collected during this research study. Your permission for the use, retention, and sharing of your identifiable health information will continue indefinitely. Any research information in your medical record will be kept indefinitely. If you choose to participate, you are free to withdraw your permission for the use and sharing of your health information at any time. You must do this in writing. Write to Dr. Sciamanna and let him know that you are withdrawing from the research study. His mailing address is:

Dr. Christopher N. Sciamanna, MD, MPH
Professor of Medicine,
Chief, Division of General Internal Medicine, HU15
Penn State Milton S. Hershey Medical Center

500 University Drive, Hershey, PA 17033

If you withdraw your permission:

- We will no longer use or share medical information about you for this research study, except when the law allows us to do so.
- We are unable to take back anything we have already done or any information we have already shared with your permission.
- We may continue using and sharing the information obtained prior to your withdrawal if it is necessary for the soundness of the overall research.
- We will keep our records of the care that we provided to you as long as the law requires.

The research team may use the following sources of health information.

- Your medical history and chart information as it relates to this research study, specifically, but not limited to: insurance billing codes, blood pressure values, intensity of treatment of hypertension during visits (e.g., medication dose changes, serum creatinine testing, urine protein testing, serum potassium testing.)
- Your chart information can be pulled beginning one year before enrollment and one year after completion of the study for the information listed above.
- All study questionnaires
- All information entered into the website

Representatives of the following people/groups within HMC/PSU may use your health information and share it with other specific groups in connection with this research study.

- The principal investigator, Christopher N. Sciamanna, MD MPH
- The HMC/PSU Institutional Review Board
- The HMC/PSU Human Subjects Protection Office
- The research team such as co-investigators, data analysts and research study coordinators

The above people/groups may share your health information with the following people/groups outside HMC/PSU for their use in connection with this research study. These groups, while monitoring the research study, may also review and/or copy your original PSU/HMC records.

- The Office of Human Research Protections in the U. S. Department of Health and Human Services
- The National Institutes of Health

8. <u>Costs for Participation:</u>

There is no cost to you for participating in this study. Some of the feedback you receive may suggest that you ask your primary care provider for certain tests or treatments (e.g., a blood test for creatinine). If your doctor chooses to order that blood test, the study will not pay for it, as it would be considered standard of care.

You may want to check with your insurance carrier prior to having certain tests or procedures done.

You will not lose any legal rights by signing this form,

9. Compensation for Participation:

You will be compensated in the form of gift cards for up to a total of $190 over the course of the study. You will receive $40 for successful completion of the baseline visit. If you come in for the baseline visit but are excluded from the study, you will receive $20. You will receive $5 for every 30-day period that they login to the website at least once. Over the next 12 months, you can receive a total possible reimbursement of $60 for logging into the website. This reimbursement is distributed to in two installments: up to $30 for the first 6, 30-day periods (i.e. after the first 180 days) which will be mailed, and up to $30 for the second 6, 30-day periods which is either given at 12-month follow up visit or mailed at the end of the study. You will also receive $10 for completion of the PCP Exit Measure, a questionnaire following your next scheduled visit with the doctor you see for hypertension care. This payment will be mailed. Lastly, you will receive $80 for successful completion of the 12-month follow-up visit.

10. Research Funding:

The institution and investigators are receiving a grant from Nation Heart, Lung and Blood Institute of the National Institutes of Health to support this research.

11. Voluntary Participation:

Taking part in this research study is voluntary. If you choose to take part in this research, your major responsibilities will include filling out questionnaires, using the website and attend two office visits. You do not have to participate in this research. If you choose to take part, you have the right to stop at any time. If you decide not to participate or if you decide to stop taking part in the research at a later date, there will be no penalty or loss of benefits to which you are entitled.

During the course of the research you will be provided with any significant new findings that may affect your willingness to continue participating in this research.

12. Contact Information for Questions or Concerns:

You have the right to ask any questions you may have about this research. If you have questions, complaints or concerns or believe you may have developed an injury related to this research, contact Dr. Christopher N. Sciamanna at 717-531-4230.

If you have questions regarding your rights as a research participant or you have concerns or general questions about the research or about your privacy and the use of your personal health information, contact the research protection advocate in the HMC

Human Subjects Protection Office at 717-531-5687. You may also call this number if you cannot reach the research team or wish to talk to someone else.

For more information about participation in a research study and about the Institutional Review Board (IRB), a group of people who review the research to protect your rights, please visit the HMC IRB's Web site at http://www.hmc.psu.edu/irb. Included on this web site, under the heading "Participant Info", you can access federal regulations and information about the protection of human research participants. If you do not have access to the internet, copies of these federal regulations are available by calling the HSPO at (717) 531-5687.

Signature and Consent/Permission to be in the Research

Before making the decision regarding enrollment in this research you should have:

- Discussed this study with an investigator
- Reviewed the information in this form, and
- Had the opportunity to ask any questions you may have.

Your signature below means that you have received this information, have asked the questions you currently have about the research and those questions have been answered. You will receive a copy of the signed and dated form to keep for future reference.

Participant: By signing this consent form, you indicate that you are voluntarily choosing to take part in this research.

______________________________	___________	_______	__________________
Signature of Participant	Date	Time	Printed Name

Person Explaining the Research: Your signature below means that you have explained the research to the participant/participant representative and have answered any questions he/she has about the research.

______________________________	___________	_______	__________________
Signature of person who explained this research	Date	Time	Printed Name

(Only approved investigators for this research may explain the research and obtain informed consent.)
