# Supplementary material for: Effects of a Web-Based Patient Activation Intervention to Overcome Clinical Inertia on Blood Pressure Control: Cluster Randomized Controlled Trial
Source: J Med Internet Res. 2013 Sep 4;15(9):e158. doi: 10.2196/jmir.2298 (PMC3785979; doi:10.2196/jmir.2298)
Supplement: Supplementary file 3 [file jmir_v15i9e158_app3.pdf]

PCP EXIT SURVEY SCREENSHOTS

myexpertdoctor®

ASK THE RIGHT QUESTIONS
GET BETTER CARE

Please select either Yes or No to indicate the topics that were discussed during your last visit with the doctor that you see for high blood pressure. If the question does not pertain to you, please select N/A. For instance, if you are a male and the question regards female health, you would select N/A.

During your last visit with the doctor that you see for your high blood pressure, did your doctor...	Yes	No	N/A
Discuss what you can do to keep your blood pressure where it is?	◉	○	○
Discuss what can be done to help lower your blood pressure?	○	◉	○
Discuss what your last blood pressure numbers were?	○	◉	○
Recommend that you START taking a new medication for your blood pressure?	○	◉	○
Recommend that you INCREASE the dose of at least one medicine you were already taking for your blood pressure?	○	◉	○
Discuss whether a routine would help you to take your medicine(s) every day?	◉	○	○
Discuss whether you might benefit from changing a blood pressure medicine to one that does not bother you?	◉	○	○
Discuss whether you might benefit from changing a blood pressure medicine to one that works better for you?	○	◉	○
Discuss whether you might benefit from changing a blood pressure medicine to one that is less expensive?	◉	○	○
Discuss whether you might benefit from changing a blood pressure medicine to one that is taken less often?	○	◉	○
Discuss whether you might benefit from having a blood test to make sure you do not have diabetes?	◉	○	○
Discuss whether you might benefit from having your cholesterol checked?	○	◉	○

PCP EXIT SURVEY SCREENSHOTS

Discuss what can be done to help you quit smoking?

Discuss what lifestyle changes would help you control your blood pressure?

Discuss whether you might benefit from being seen more reqularly until your blood pressure is controlled?

Discuss whether you might benefit from being tested to see if something is causing your blood pressure to be so high?

Discuss whether you are due to have a blood test for creatinine? This test measures your kidney function.

Discuss the results of your last creatinine blood test?

Discuss whether you might benefit from having your urine tested for protein?

Discuss whether you might benefit from seeing a blood pressure specialist? These specialists are also called cardiologists or nephrologists.

Discuss what you can do to keep your kidneys healthy?

Discuss whether your kidneys may be damaged?

Discuss whether you should have a test for colon cancer?

Discuss whether you should have a mammogram?

Discuss whether you should have a Pap test? A Pap test is a test for cancer of the cervix.

Discuss whether you should have a flu shot?

Discuss whether you should have a pneumonia shot? This shot is usually given only once or twice in a person's lifetime and is different from the flu shot. It is also called the pneumococcal vaccine.

Discuss whether you should have a tetanus shot?

PCP EXIT SURVEY SCREENSHOTS

Additional questions concerning your visit.	Yes	No	N/A
Did you print any of the pages of this website?	◉	○	○
Did you make handwritten notes about any of the pages of this website?	○	◉	○
Did you bring handwritten notes or any printed pages of this website to the doctor visit?	◉	○	○
Did you show your doctor any handwritten notes or printed pages of this website?	◉	○	○
Did you ask any specific questions from this website during the doctor visit?	◉	○	○
After your blood pressure was taken, did you write down the blood pressure numbers?	◉	○	○

Save and Continue
