# Supplementary material for: Effects of a Web-Based Patient Activation Intervention to Overcome Clinical Inertia on Blood Pressure Control: Cluster Randomized Controlled Trial
Source: J Med Internet Res. 2013 Sep 4;15(9):e158. doi: 10.2196/jmir.2298 (PMC3785979; doi:10.2196/jmir.2298)
Supplement: Supplementary file 4 [file jmir_v15i9e158_app4.pdf]

ONLINE FOLLOW-UP MEASURES

myexpertdoctor®

ASK THE RIGHT QUESTIONS
GET BETTER CARE

About how much do you weigh without shoes (in pounds)?

Weight: 158

	Yes	No
**Are you now trying to lose weight?**	◉	○

	Yes	No
**Do you have any kind of health care coverage?**	◉	○
**Was there a time in the past 12 months when you needed medical care, but could not get it?**	○	◉

How would describe your general health? Poor

Now thinking about your physical health, which includes physical illness and injury, for how many days during the past 30 days was your physical health NOT GOOD?

Days: 0

Now thinking about your mental health, which includes stress, depression and problems with emotions, for how many days during the past 30 days was your mental health NOT GOOD?

Days: 0

ONLINE FOLLOW-UP MEASURES

Within the past 12 months, has a doctor, nurse, or other health professional advised you to:

	Yes	No
**Eat fewer high fat or high cholesterol foods?**	◉	○
**Eat more fruits and vegetables?**	○	◉
**Lose weight?**	◉	○
**Be more physically active?**	○	◉
**Quit smoking?**	◉	○
**Drink less alcohol?**	○	◉

Percent Complete:

Next Segment

(Please just click *once*. The system may take a few moments to respond to your request.)

Any missing or incomplete answers will be marked with RED above.

ONLINE FOLLOW-UP MEASURES

myexpertdoctor®

ASK THE RIGHT QUESTIONS
GET BETTER CARE

How stongly do you AGREE or DISAGREE with the following statements:

My doctor is good at explaining the reason for medical tests.	Disagree
My doctor's office has everything needed to provide complete medical care.	Uncertain
Sometimes my doctor makes me wonder if his/her diagnoses are correct.	Disagree
When I go for medical care, my doctor is careful to check everything when treating and examining me.	Strongly disagree
My doctor acts too businesslike and impersonal toward me.	Disagree
My doctor treats me in a very friendly and courteous manner.	Uncertain
My doctor sometimes hurries too much when treating me.	Uncertain
My doctor sometimes ignores what I say.	Uncertain
I have some doubts about the ability of my doctor.	Uncertain
My doctor usually spends plenty of time with me.	Uncertain
I trust my doctor.	Uncertain

Please choose the most appropriate response for each of the following:

**How often DO YOU ASK YOUR DOCTOR to explain your treatment options?**	Always
**How often DO YOU ASK YOUR DOCTOR to help you understand what he/she tells you?**	Usually
**How often DO YOU ASK YOUR DOCTOR about the possible side effects of treatments?**	Usually
**How often DO YOU ASK YOUR DOCTOR for the results of tests you had?**	Sometimes
**How often do you write down your test results or ask for a copy?**	Sometimes

Percent Complete:

Next Segment

(Please just click *once*. The system may take a few moments to respond to your request.)

Any missing or incomplete answers will be marked with RED above.

ONLINE FOLLOW-UP MEASURES

myexpertdoctor®

ASK THE RIGHT QUESTIONS
GET BETTER CARE

How stongly do you AGREE or DISAGREE with the following statements?

When all is said and done, I am the person who is responsible for managing my health condition.	Disagree strongly
Taking an active role in my own health care is the most important factor in determining my health and ability to function.	Disagree
I am confident that I can take actions that will help prevent or minimize some symptoms or problems associated with my health condition.	Agree
I know what each of my prescribed medications does.	Agree strongly
I am confident that I can tell when I need to go get medical care and when I can handle a health problem myself.	N/A
I am confident I can tell a doctor concerns I have even when he or she does not ask.	Disagree strongly
I am confident that I can follow through on medical treatments I need to do at home.	Disagree
I understand the nature and causes of my health condition(s).	Agree

ONLINE FOLLOW-UP MEASURES

**I know the different medical treatment options available for my health condition.**	Agree strongly
**I have been able to maintain the lifestyle changes for my health condition that I have made.**	N/A
**I know how to prevent further problems with my health condition.**	Disagree
**I am confident I can figure out solutions when new situations or problems arise with my health condition.**	Agree
**I am confident that I can maintain lifestyle changes, like diet and exercise even during times of stress.**	Agree strongly

Percent Complete:

Next Segment

(Please just click *once*. The system may take a few moments to respond to your request.)

Any missing or incomplete answers will be marked with RED above.

ONLINE FOLLOW-UP MEASURES

myexpertdoctor®

ASK THE RIGHT QUESTIONS
GET BETTER CARE

	Yes	No	Not Sure
**Do you ever go online to access the Internet (or World Wide Web) or to send and receive email?**	(•)	( )	( )

How often do you use the Internet to look for advice or information about health or health care? [Several times a month]

	Yes	No	Don't know
**Have you ever used the Internet to look for information about a particular illness or condition?**	(•)	( )	( )
**Have you ever used the Internet to look for information about a mental health issue like depression or anxiety?**	( )	(•)	( )
**Have you ever used the Internet to look for information about a particular doctor or hospital?**	( )	( )	(•)
**Have you ever used the Internet to look for information about alternative or experimental treatments or medicines?**	(•)	( )	( )
**Have you ever used the Internet to look for information about a sensitive health topic that is difficult to talk about?**	( )	(•)	( )
**Have you ever used the Internet to look for information or advice about nutrition, exercise, or weight control?**	( )	( )	(•)
**Have you ever used the Internet to look for information about prescription drugs?**	(•)	( )	( )
**Have you ever used the Internet to diagnose or treat a medical condition on your own, without consulting your doctor?**	( )	(•)	( )
**Have you ever used the Internet to gather information BEFORE visiting your doctor?**	( )	( )	(•)
**Have you ever emailed your doctor's office with a medical question?**	(•)	( )	( )

Percent Complete:

[Next Segment]

(Please just click *once*. The system may take a few moments to respond to your request.)

Any missing or incomplete answers will be marked with RED above.

ONLINE FOLLOW-UP MEASURES

myexpertdoctor®

ASK THE RIGHT QUESTIONS
GET BETTER CARE

	Yes	No
Do you smoke cigarettes?	◉	○

	Yes	No
Have you smoked 100 or more cigarettes during your lifetime?		

When was the last time you had a flu shot?

If you are not exactly sure, please make your best guess. If you have never had a flu shot, please leave blank.

Month: 11 Year: 2007

When was the last time you had a pneumonia vaccination?

If you are not exactly sure, please make your best guess. If you have never had a pneumonia vaccination, please leave blank.

Month: 8 Year: 2001

When was the last time you had a tetanus shot?

If you are not exactly sure, please make your best guess. If you have never had a tetanus shot, please leave blank.

Month: 7 Year: 2000

	Yes	No
Do you take aspirin daily or every other day?	◉	○

	Yes	No	Not Sure
Have you ever been told by a doctor that you have asthma, emphysema, chronic bronchitis, or chronic obstructive pulmonary disease?	○	◉	○
Has a doctor ever told you that you had a heart attack, angina, or coronary heart disease?	○	◉	○

A mammogram is an x-ray of each breast to look for breast cancer. When was the last time you had a mammogram?

If you are not exactly sure, please make your best guess. If you have never had a mammogram, please leave blank.

Month: 10 Year: 1999

ONLINE FOLLOW-UP MEASURES

A Pap test is a test for cervical cancer. When was the last time you had a Pap test?

If you are not exactly sure, please make your best guess. If you have never had a pap test, please leave blank.

Month: 6 Year: 2007

Have you gone through or are you now going through menopause, also know as the "change of life"?

- Yes, I have gone through menopause ○
- Yes, I am currently going through menopause ⊙
- No ○

	Yes	No	Not Sure
Since the age of 18, have you ever fractured a bone?	⊙	○	○

Did that fracture occur as a result of a fall from standing height or less, did it occur because of a harder fall, or did it occur from a car accident or other severe trauma?

- Standing height or less ○
- Harder fall ⊙
- Severe trauma ○
- Not Sure ○

	Yes	No	Not Sure
Has your mother or father ever broken or fractured their hip?	○	⊙	○

Bone density is measured using a test called a "DXA" or "DEXA" scan. The test is typically done on a bone in your hip, spine, or wrist. DEXA scans of the wrist, however, are less accurate. When was the last time you had your bone density measured in your hip or spine?

If you are not exactly sure, please make your best guess. If you have never had a bone density test, please leave blank.

Month: 3 Year: 2006

A blood stool test is a test in which a special kit is used at home to determine whether the stool contains blood. When was the last time you had a blood stool test?

If you are not exactly sure, please make your best guess. If you have never had a blood stool test, please leave blank.

Month: -- Year: 1989

A barium enema is a test in which a milky fluid is inserted in the rectum and an x-ray is taken of the intestines. When was the last time you had a barium

A barium enema is a test in which a milky fluid is inserted in the rectum and an x-ray is taken of the intestines. When was the last time you had a barium enema?

If you are not exactly sure, please make your best guess. If you have never had a barium enema, please leave blank.

Month: 4 **Year:** --

A sigmoidoscopy is a test in which a tube is inserted in the rectum to view the bowel for signs of cancer and other health problems. When was the last time you had a sigmoidoscopy?

If you are not exactly sure, please make your best guess. If you have never had a sigmoidoscopy, please leave blank.

Month: 10 **Year:** 1991

A colonoscopy is another test in which a tube is inserted in the rectum to view the bowel for signs of cancer and other health problems. When was the last time you had a colonoscopy?

If you are not exactly sure, please make your best guess. If you have never had a colonoscopy, please leave blank.

Month: 2 **Year:** 1989

Considering all types of alcoholic beverages, how many times during the past month did you have 4 or more drinks on an occasion?

(If you would rather not answer, please leave the answer blank.)

Answer: 17

During the past month, how many times have you driven when you've had too much to drink?

(If you would rather not answer, please leave the answer blank.)

Answer: 5

Percent Complete:

Next Segment

(Please just click *once*. The system may take a few moments to respond to your request.)

Any missing or incomplete answers will be marked with RED above.

ONLINE FOLLOW-UP MEASURES

myexpertdoctor®

ASK THE RIGHT QUESTIONS
GET BETTER CARE

	Yes	No	Not Sure
Are you currently being treated for diabetes? Learn More	○	⦿	○

	Yes	No	Not sure
Have you ever been told by a doctor that you have chronic kidney disease?	○	⦿	○

A blood test for creatinine measures your kidney function. When was the last time you had your creatinine checked? Learn More

If you are not exactly sure, please make your best guess. If you have never had your creatinine checked, please leave blank.

Month: 2 Year: 1995

What was the value of your last creatinine test?

If you do not remember, or have never had your creatinine checked, please leave blank.

Result: 2.1

When was the last time you had your urine tested for protein? Learn More

If you are not exactly sure, please make your best guess. If you have never had your urine tested, please leave blank.

Month: 3 Year: 1988

When was the last time you had your cholesterol checked? Learn More

If you are not exactly sure, please make your best guess. If you have never had your cholesterol checked, please leave blank.

Month: 3 Year: --

ONLINE FOLLOW-UP MEASURES

When was the last time your doctor checked your blood pressure?

If you are not exactly sure, please make your best guess. If you have never had your blood pressure checked, please leave blank.

Month: [--] **Year:** [1991]

What was your blood pressure reading the last time you had it checked?

If you do not remember, or if you have never had your blood pressure checked, please leave blank.

Systolic (top number): 156

Diastolic (bottom number): 98

	Yes	No	Not Sure
**Do you take any medicines to control your blood pressure?**	⦿	○	○

Please consider your blood pressure medicines when answering the following questions.

	Yes	No
**Do you ever forget to take your medications?**	○	⦿
**Are you careless at times about taking your medications?**	○	⦿
**When you feel better, do you sometimes stop taking your medications?**	○	⦿
**Sometimes if you feel worse when you take your medications, do you stop taking them?**	○	⦿
**Do you have a routine for taking your blood pressure medicines the same way each day?**	⦿	○
**Do any of your blood pressure medicines bother you in any way?**	⦿	○
**Do you ever have trouble paying for your blood pressure medicines?**	⦿	○
**Do you take any blood pressure medicines more than two times each day?**	⦿	○

Are any of your blood pressure medicines NOT working well?

Yes, one or more of my medicines is not working well: ○
No, all of my blood pressure medicines are working well: ⊙

When was the last time you had a discussion with a doctor about lifestyle changes that could help you to control your blood pressure? Learn More

If you are not exactly sure, please make your best guess. If you have never discussed this with your doctor, please leave blank.

Date of last discussion: Month: 4 **Year:** --

When was the last time you saw a blood pressure specialist? Learn More
These specialists are also called cardiologists or nephrologists.

If you are not exactly sure, please make your best guess. If you have never seen a specialist, please leave blank.

Month: -- **Year:** 1989

	Yes	No
**Would you like to participate in similar studies in the future?**	○	○

Percent Complete:

Next Segment

(Please just click *once*. The system may take a few moments to respond to your request.)

Any missing or incomplete answers will be marked with RED above.
