# Supplementary material for: Effects of a Web-Based Patient Activation Intervention to Overcome Clinical Inertia on Blood Pressure Control: Cluster Randomized Controlled Trial
Source: J Med Internet Res. 2013 Sep 4;15(9):e158. doi: 10.2196/jmir.2298 (PMC3785979; doi:10.2196/jmir.2298)
Supplement: Supplementary file 5 [file jmir_v15i9e158_app5.pdf]

INTERVENTION MONTHLY SURVEY

myexpertdoctor®

ASK THE RIGHT QUESTIONS
GET BETTER CARE

Please review your responses below. If your answers have changed since your last visit to the website, please correct your responses appropriately. If your answers have not changed since your last visit to the website, please verify that the responses are still accurate.

Clicking the 'Submit and Continue' button at the bottom of the screen will save your answers, and ensure that you receive credit for using the site.

For each answer requiring a month and year, please use the drop down list to select the month and year to the best of your knowledge. Your responses will help us to suggest questions that you can ask your doctor at your next visit.

	Yes	No
**Do you smoke cigarettes?**	◉	○

	Yes	No	Not Sure
**Are you currently being treated for diabetes?** Learn More	○	◉	○

	Yes	No	Not sure
**Have you ever been told by a doctor that you have chronic kidney disease?**	○	◉	○

A blood test for creatinine measures your kidney function. When was the last time you had your creatinine checked? Learn More

If you are not exactly sure, please make your best guess. If you have never had your creatinine checked, please leave blank.

Month: 4 **Year:** 2008

What was the value of your last creatinine test?

If you do not remember, or have never had your creatinine checked, please leave blank.

Result: --

When was the last time you had your urine tested for protein? Learn More

If you are not exactly sure, please make your best guess. If you have never had your urine tested, please leave blank.

Month: 3 **Year:** 2004

When was the last time you had your cholesterol checked? Learn More

If you are not exactly sure, please make your best guess. If you have never had your cholesterol checked, please leave blank.

Month: 3 **Year:** 2005

When was the last time your doctor checked your blood pressure?

If you are not exactly sure, please make your best guess. If you have never had your blood pressure checked, please leave blank.

Month: 5 **Year:** 2010

What was your blood pressure reading the last time you had it checked?

If you do not remember, or if you have never had your blood pressure checked, please leave blank.

Systolic (top number): 122

Diastolic (bottom number): 72

	Yes	No	Not Sure
**Do you take any medicines to control your blood pressure?**	◉	○	○

Please answer the following questions about your blood pressure medicine:

	Yes	No
**Do you have a routine for taking your blood pressure medicines the same way each day?**	◉	○
**Do any of your blood pressure medicines bother you in any way?**	◉	○
**Do you ever have trouble paying for your blood pressure medicines?**	◉	○
**Do you take any blood pressure medicines more than two times each day?**	○	◉

Are any of your blood pressure medicines NOT working well?

Yes, one or more of my medicines is not working well: ○

No, all of my blood pressure medicines are working well: ○

When was the last time you had a discussion with a doctor about lifestyle changes that could help you to control your blood pressure? Learn More

If you are not exactly sure, please make your best guess. If you have never discussed this with your doctor, please leave blank.

Date of last discussion: Month: 4 **Year:** 2003

When was the last time you saw a blood pressure specialist? Learn More
These specialists are also called cardiologists or nephrologists.

If you are not exactly sure, please make your best guess. If you have never seen a specialist, please leave blank.

Month: 3 **Year:** 2008

Please tell us the date for your next scheduled visit with the doctor you see for your blood pressure. If you see more than one doctor for your blood pressure, please tell us the date of the visit coming soonest.

If you are not sure of the date, please estimate. You will be able to change this date later if you need to.

Enter Next Scheduled Visit Date (mm/dd/yy): 08/18/10

Submit and Continue

(Please just click *once*. The system may take a few moments to respond to your request.)

Any missing or incomplete answers will be marked with RED above.
