# Supplementary material for: Effects of a Web-Based Patient Activation Intervention to Overcome Clinical Inertia on Blood Pressure Control: Cluster Randomized Controlled Trial
Source: J Med Internet Res. 2013 Sep 4;15(9):e158. doi: 10.2196/jmir.2298 (PMC3785979; doi:10.2196/jmir.2298)
Supplement: Supplementary file 6 [file jmir_v15i9e158_app6.pdf]

INTERVENTION FEEDBACK

IT'S ALL ABOUT THE NUMBERS!

Thanks again for your help in this important study! Your job in this study is to speak up and ask questions during your doctor visits. We will NOT send any of this information to your doctor or contact you about it. It is up to YOU to discuss these issues with your doctor. It is your doctor's job to keep making changes in your care until you reach the goal for both blood pressure numbers.

Click for more information

HOW'S MY BLOOD PRESSURE?

For all patients with high blood pressure, closely controlling your numbers is essential. This includes both your systolic blood pressure (top number) and diastolic blood pressure (bottom number).

Systolic Blood Pressure (top number).
Your goal: a number less than 140.
Your last reading was 122.

Diastolic Blood Pressure (bottom number).
Your goal: a number less than 90.
Your last reading was 72.

THE MOST IMPORTANT QUESTIONS TO ASK YOUR DOCTOR.

WHAT CAN I DO TO KEEP MY BLOOD PRESSURE WHERE IT IS?

Congratulations! Your blood pressure is nicely controlled!

Keeping your systolic blood pressure (top number) less than 140 and your diastolic blood pressure (bottom number) less than 90 is a great way to prevent heart attacks and strokes.

Keep up the good work!

Click for more information

SHOULD A BLOOD PRESSURE MEDICINE BE CHANGED TO ONE THAT DOESN'T BOTHER ME?

From what you've told us, a blood pressure medicine may be bothering you. It is often hard to know what is causing bothersome symptoms. They may or may not be from a medicine. Your doctor may do one of the following:

--Change the *dose* of a medicine
--Change you to a *different* medicine
--Change *how* or *when* you take a medicine

Click for more information

SHOULD I HAVE A BLOOD TEST TO MAKE SURE I DON'T HAVE DIABETES?

People with high blood pressure are at high risk for diabetes. For that reason, patients with high blood pressure should regularly be checked for diabetes. This can be done using a blood test, typically done in your doctor's office. You should talk to your doctor about this.

Click for more information

SHOULD I HAVE MY CHOLESTEROL CHECKED?

People with high blood pressure are at risk for having high cholesterol. For that reason, patients with high blood pressure should have their cholesterol checked regularly. This can be done using a blood test, typically done in your doctor's office. You should talk to your doctor about this.

Click for more information

WHAT CAN YOU DO TO HELP ME QUIT SMOKING?

People who smoke and have high blood pressure are at very high risk of having a heart attack or stroke. You should consider setting a date to quit smoking and think about using a medication to help you. Nicotine patches, gum, Zyban and Chantix are all effective at helping smokers quit. Nicotine patches and gum are available without a prescription, but you'll need a prescription from your doctor for Zyban and Chantix.

To speak with someone other than a doctor who can help you quit, call the American Cancer Society toll-free at 1-800-ACS-2345.

Click for more information

WHAT LIFESTYLE CHANGES WOULD HELP TO CONTROL MY BLOOD PRESSURE?

Lifestyle changes can help people lower their blood pressure. Weight loss, regular exercise and eating less salt, more fruits, vegetables and low-fat dairy products have all been proven to help. You should talk to your doctor about which of these changes may be most helpful for you.

Click for more information

AM I DUE TO HAVE A BLOOD TEST FOR CREATININE AND POTASSIUM?

At least once every year, people with high blood pressure should have a blood test for creatinine and potassium. People with high blood pressure are at high risk for

INTERVENTION FEEDBACK

Click for more information

AM I DUE TO HAVE A BLOOD TEST FOR CREATININE AND POTASSIUM?

At least once every year, people with high blood pressure should have a blood test for creatinine and potassium. People with high blood pressure are at high risk for kidney damage. People with more creatinine and potassium in the blood need to have their blood pressure controlled more closely.

Click for more information

WHAT WAS THE RESULT OF MY LAST CREATININE BLOOD TEST?

We're not sure about your kidney function because you didn't enter a creatinine blood test result. You may want to ask your doctor for your test results. To help with this, you may want to print this page and bring it to your next doctor visit, so you can write down your creatinine blood test results. Creatinine is a commonly done test of kidney function. A normal value is less than 1.5 for a man and less than 1.3 for a woman.

Click for more information

SHOULD I HAVE MY URINE TESTED FOR PROTEIN?

From what you've told us, you have not had a urine protein test in the past year. You may want to ask your doctor about this. Many people with high blood pressure can benefit from having a yearly test to check for protein in their urine. High blood pressure can damage the kidneys, so checking the level of protein in the urine helps your doctor know if your blood pressure is controlled well enough.

Click for more information

What information should I collect from my doctor?

Test Results	Last Result	Date	New Result	Date
Blood Pressure	122 / 72	5/2010	___ / ___	___/___/___
Creatinine Test	N/A	4/2008	___ / ___	___/___/___
Urine Protein Test	________	3/2004	___ / ___	___/___/___

Notes

INTERVENTION FEEDBACK

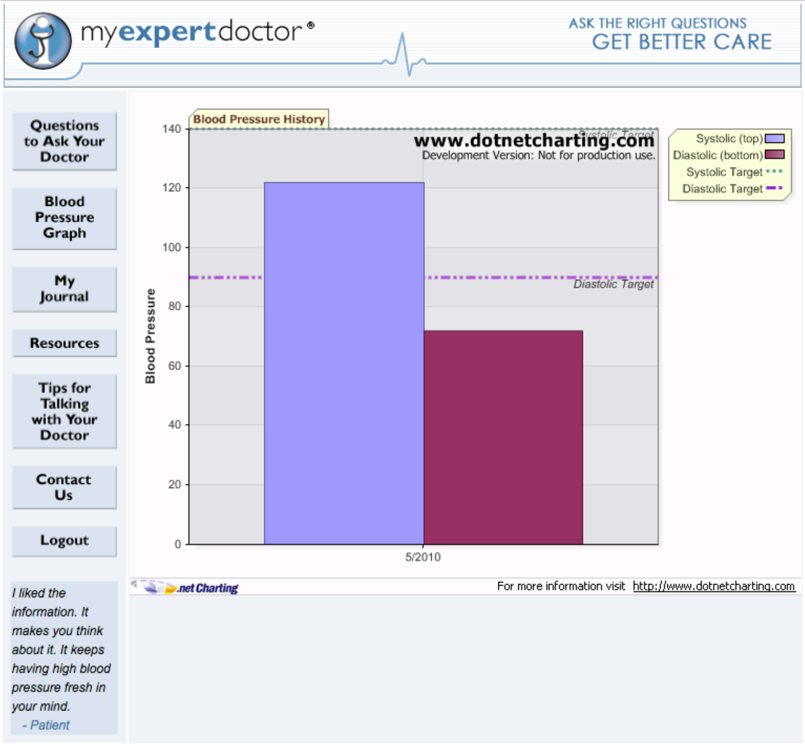

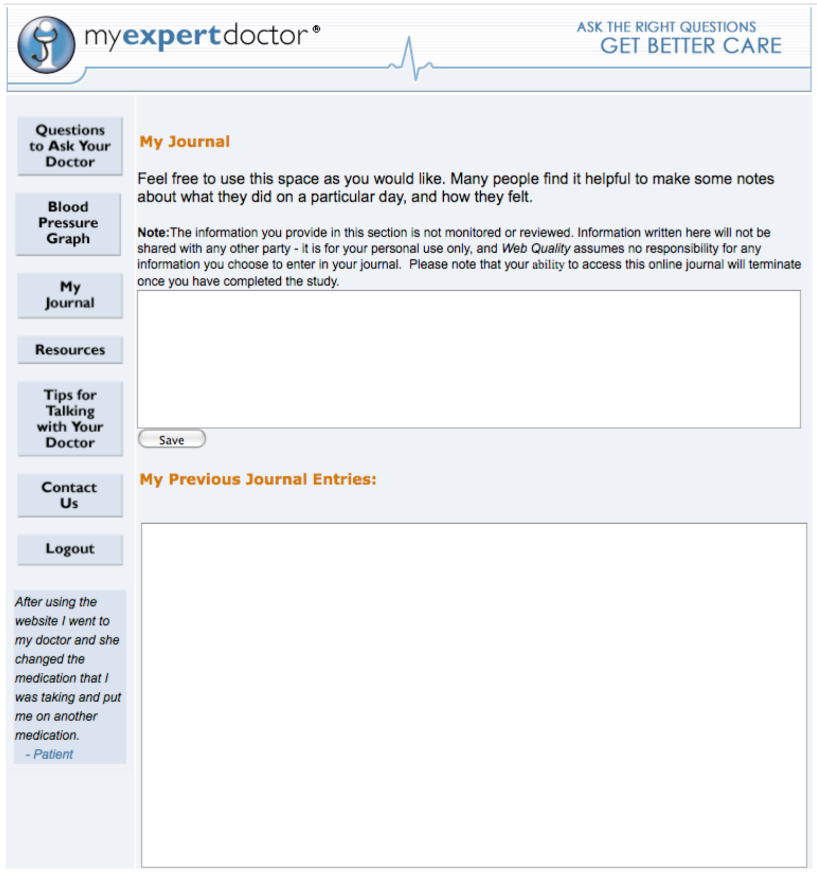

INTERVENTION FEEDBACK

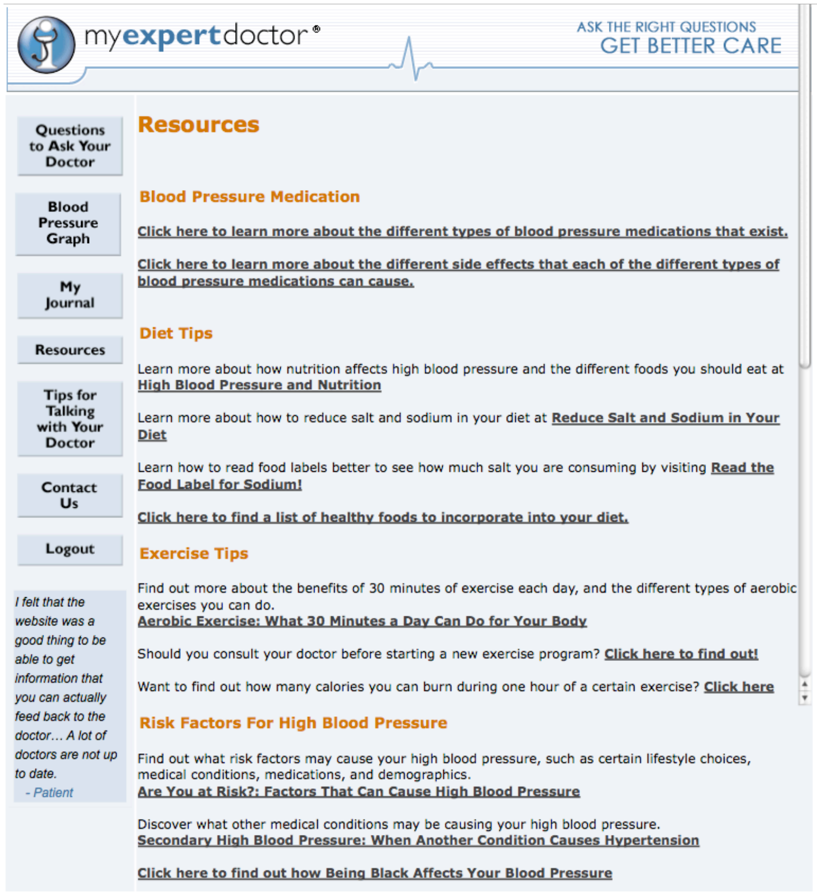

myexpertdoctor®

ASK THE RIGHT QUESTIONS
GET BETTER CARE

Questions to Ask Your Doctor
Blood Pressure Graph
My Journal
Resources
Tips for Talking with Your Doctor
Contact Us
Logout

I felt that the website was a good thing to be able to get information that you can actually feed back to the doctor… A lot of doctors are not up to date.
- Patient

Resources

Blood Pressure Medication

Click here to learn more about the different types of blood pressure medications that exist.

Click here to learn more about the different side effects that each of the different types of blood pressure medications can cause.

Diet Tips

Learn more about how nutrition affects high blood pressure and the different foods you should eat at **High Blood Pressure and Nutrition**

Learn more about how to reduce salt and sodium in your diet at **Reduce Salt and Sodium in Your Diet**

Learn how to read food labels better to see how much salt you are consuming by visiting **Read the Food Label for Sodium!**

Click here to find a list of healthy foods to incorporate into your diet.

Exercise Tips

Find out more about the benefits of 30 minutes of exercise each day, and the different types of aerobic exercises you can do.
Aerobic Exercise: What 30 Minutes a Day Can Do for Your Body

Should you consult your doctor before starting a new exercise program? **Click here to find out!**

Want to find out how many calories you can burn during one hour of a certain exercise? **Click here**

Risk Factors For High Blood Pressure

Find out what risk factors may cause your high blood pressure, such as certain lifestyle choices, medical conditions, medications, and demographics.
Are You at Risk?: Factors That Can Cause High Blood Pressure

Discover what other medical conditions may be causing your high blood pressure.
Secondary High Blood Pressure: When Another Condition Causes Hypertension

Click here to find out how Being Black Affects Your Blood Pressure

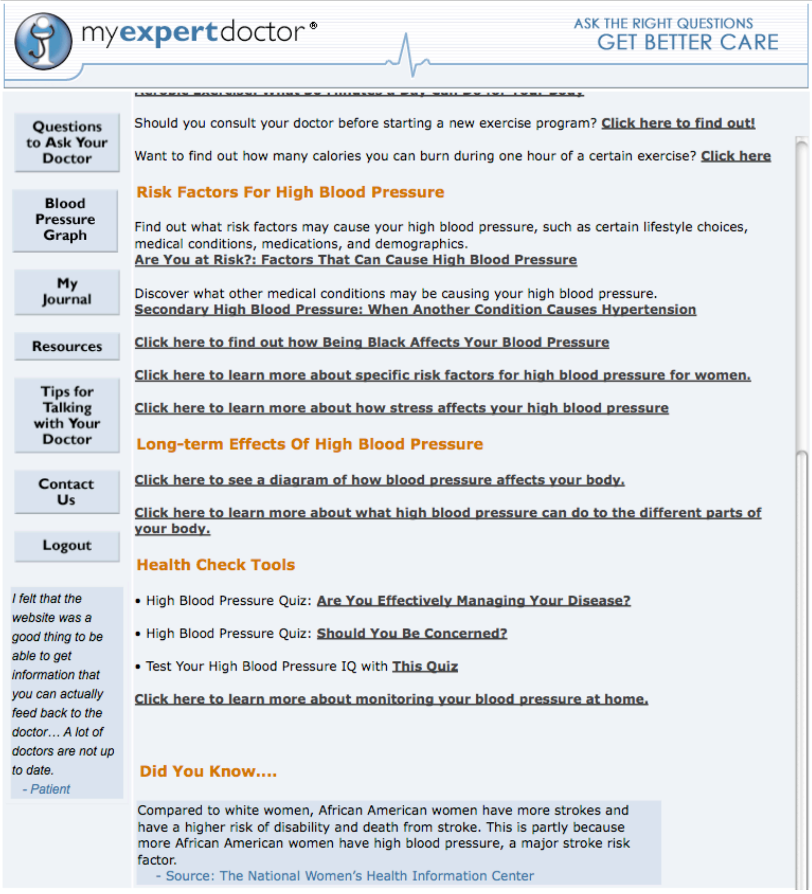

myexpertdoctor®

ASK THE RIGHT QUESTIONS
GET BETTER CARE

Questions to Ask Your Doctor
Blood Pressure Graph
My Journal
Resources
Tips for Talking with Your Doctor
Contact Us
Logout

I felt that the website was a good thing to be able to get information that you can actually feed back to the doctor… A lot of doctors are not up to date.
- Patient

[illegible]

Should you consult your doctor before starting a new exercise program? **Click here to find out!**

Want to find out how many calories you can burn during one hour of a certain exercise? **Click here**

Risk Factors For High Blood Pressure

Find out what risk factors may cause your high blood pressure, such as certain lifestyle choices, medical conditions, medications, and demographics.
Are You at Risk?: Factors That Can Cause High Blood Pressure

Discover what other medical conditions may be causing your high blood pressure.
Secondary High Blood Pressure: When Another Condition Causes Hypertension

Click here to find out how Being Black Affects Your Blood Pressure

Click here to learn more about specific risk factors for high blood pressure for women.

Click here to learn more about how stress affects your high blood pressure

Long-term Effects Of High Blood Pressure

Click here to see a diagram of how blood pressure affects your body.

Click here to learn more about what high blood pressure can do to the different parts of your body.

Health Check Tools

- High Blood Pressure Quiz: **Are You Effectively Managing Your Disease?**
- High Blood Pressure Quiz: **Should You Be Concerned?**
- Test Your High Blood Pressure IQ with **This Quiz**

Click here to learn more about monitoring your blood pressure at home.

Did You Know….

Compared to white women, African American women have more strokes and have a higher risk of disability and death from stroke. This is partly because more African American women have high blood pressure, a major stroke risk factor.
- Source: The National Women's Health Information Center

INTERVENTION FEEDBACK

myexpertdoctor®

ASK THE RIGHT QUESTIONS
GET BETTER CARE

- Questions to Ask Your Doctor
- Blood Pressure Graph
- My Journal
- Resources
- Tips for Talking with Your Doctor
- Contact Us
- Logout

I felt that the website was a good thing to be able to get information that you can actually feed back to the doctor… A lot of doctors are not up to date.
- Patient

Tips For Talking with Your Doctor

The single most important way you can stay healthy is to be an active member of your own health care team. One way to get high-quality health care is to find and use information and take an active role in all of the decisions made about your care.

This information will help you when talking with your doctor.

Research has shown that patients who have good relationships with their doctors tend to be more satisfied with their care—and tend to have better results. Here are some tips to help you and your doctor become partners in improving your health care.

GIVE INFORMATION. DON'T WAIT TO BE ASKED!

- You know important things about your symptoms and your health history. Tell your doctor what you think he or she needs to know.
- It is important to tell your doctor personal information—even if it makes you feel embarrassed or uncomfortable.
- Bring a "health history" list with you, and keep it up to date. A "health history" is a list that includes any illnesses, diagnoses, tests, and treatments that you've had and any medications that you've taken. You might want to make a copy of the form for each member of your family.
- Always bring any medicines you are taking or a list of those medicines (include when and how often you take them) and what strength. Talk about any allergies or reactions you have had to your medicines.
- Tell your doctor about any herbal products you use or alternative medicines or treatments you receive.
- Bring other medical information, such as x-ray films, test results, and medical records.

GET INFORMATION

- Ask questions. If you don't, your doctor may think you understand everything that was said.
- Write down your questions before your visit. List the most important ones first to make sure they get asked and answered.
- You might want to bring someone along to help you ask questions. This person can also

myexpertdoctor®

ASK THE RIGHT QUESTIONS
GET BETTER CARE

GET INFORMATION

- Ask questions. If you don't, your doctor may think you understand everything that was said.
- Write down your questions before your visit. List the most important ones first to make sure they get asked and answered.
- You might want to bring someone along to help you ask questions. This person can also help you understand and/or remember the answers.
- Ask your doctor to draw pictures if that might help to explain something.

- Questions to Ask Your Doctor
- Blood Pressure Graph
- My Journal
- Resources
- Tips for Talking with Your Doctor
- Contact Us
- Logout

I felt that the website was a good thing to be able to get information that you can actually feed back to the doctor… A lot of doctors are not up to date.
- Patient

WHAT IF MY DOCTOR SAYS "NO" TO WHAT I ASK?

- Be patient and listen. Remember that your doctor knows quite a bit about you and may have a good reason for not going along with your plan.
- Ask your doctor "why not"? Your doctor may have a very good point, something that you may not have considered.
- Ask your doctor what might happen that would change his or her mind? Doctors often don't discuss a long-term plan, though they may know it. Your doctor may just be waiting a little while before making the change you asked for.
- Be persistent. Doctors tend to be less aggressive in treating chronic illnesses, such as high blood pressure. You may need to ask again at a later time.

TAKE NOTES

- Some doctors do not mind if you bring a tape recorder to help you remember things. But always ask first.
- Let your doctor know if you need more time. If there is not time that day, perhaps you can speak to a nurse or physician assistant on staff. Or, ask if you can call later to speak with someone.

TAKE INFORMATION HOME

- Ask for written instructions.
- Your doctor also may have brochures, audio tapes, and videotapes that can help you. If not, ask how you can get such materials.

ONCE YOU LEAVE THE DOCTOR'S OFFICE, FOLLOW UP

- If you have questions, call
