# Supplementary material for: Effects of a Web-Based Patient Activation Intervention to Overcome Clinical Inertia on Blood Pressure Control: Cluster Randomized Controlled Trial
Source: J Med Internet Res. 2013 Sep 4;15(9):e158. doi: 10.2196/jmir.2298 (PMC3785979; doi:10.2196/jmir.2298)
Supplement: Supplementary file 7 [file jmir_v15i9e158_app7.pdf]

PREVENTIVE MONTHLY SURVEY

myexpertdoctor®

ASK THE RIGHT QUESTIONS
GET BETTER CARE

Please review your responses below. If your answers have changed since your last visit to the website, please correct your responses appropriately. If your answers have not changed since your last visit to the website, please verify that the responses are still accurate.

Clicking the 'Submit and Continue' button at the bottom of the screen will save your answers, and ensure that you receive credit for using the site.

For each answer requiring a month and year, please use the drop down list to select the month and year to the best of your knowledge. Your responses will help us to suggest questions that you can ask your doctor at your next visit.

What is your weight (in pounds)? **Weight:** 158

	Yes	No
**Do you smoke cigarettes?**	⦿	○

Yes No

Have you smoked 100 or more cigarettes during your lifetime?

When was the last time you had a flu shot?

If you are not exactly sure, please make your best guess. If you have never had a flu shot, please leave blank.

Month: 11 **Year:** 2007

When was the last time you had a pneumonia vaccination?

If you are not exactly sure, please make your best guess. If you have never had a pneumonia vaccination, please leave blank.

Month: 8 **Year:** 2001

When was the last time you had a tetanus shot?

If you are not exactly sure, please make your best guess. If you have never had a tetanus shot, please leave blank.

Month: 7 **Year:** 2000

	Yes	No
**Do you take aspirin daily or every other day?**	⦿	○

PREVENTIVE MONTHLY SURVEY

	Yes	No	Not Sure
Have you ever been told by a doctor that you have asthma, emphysema, chronic bronchitis, or chronic obstructive pulmonary disease?	○	⊙	○
Has a doctor ever told you that you had a heart attack, angina, or coronary heart disease?	○	⊙	○
Has a doctor told you more than once that you have high blood pressure?	○	⊙	○
Have you ever been told by a doctor that you have diabetes?	⊙	○	○

A mammogram is an x-ray of each breast to look for breast cancer. When was the last time you had a mammogram?

If you are not exactly sure, please make your best guess. If you have never had a mammogram, please leave blank.

Month: 10 Year: 1999

A Pap test is a test for cervical cancer. When was the last time you had a Pap test?

If you are not exactly sure, please make your best guess. If you have never had a pap test, please leave blank.

Month: 6 Year: 2007

Have you gone through or are you now going through menopause, also know as the "change of life"?

Yes, I have gone through menopause ○
Yes, I am currently going through menopause ⊙
No ○

	Yes	No	Not Sure
Since the age of 18, have you ever fractured a bone?	⊙	○	○

Did that fracture occur as a result of a fall from standing height or less, did it occur because of a harder fall, or did it occur from a car accident or other severe trauma?

Standing height or less ○
Harder fall ⊙
Severe trauma ○
Not Sure ○

PREVENTIVE MONTHLY SURVEY

	Yes	No	Not Sure
Has your mother or father ever broken or fractured their hip?	○	⦿	○

Bone density is measured using a test called a "DXA" or "DEXA" scan. The test is typically done on a bone in your hip, spine, or wrist. DEXA scans of the wrist, however, are less accurate. When was the last time you had your bone density measured in your hip or spine?

If you are not exactly sure, please make your best guess. If you have never had a bone density test, please leave blank.

Month: 3 Year: 2006

A blood stool test is a test in which a special kit is used at home to determine whether the stool contains blood. When was the last time you had a blood stool test?

If you are not exactly sure, please make your best guess. If you have never had a blood stool test, please leave blank.

Month: -- Year: 1989

A barium enema is a test in which a milky fluid is inserted in the rectum and an x-ray is taken of the intestines. When was the last time you had a barium enema?

If you are not exactly sure, please make your best guess. If you have never had a barium enema, please leave blank.

Month: 4 Year: --

A sigmoidoscopy is a test in which a tube is inserted in the rectum to view the bowel for signs of cancer and other health problems. When was the last time you had a sigmoidoscopy?

If you are not exactly sure, please make your best guess. If you have never had a sigmoidoscopy, please leave blank.

Month: 10 Year: 1991

A colonoscopy is another test in which a tube is inserted in the rectum to view the bowel for signs of cancer and other health problems. When was the last time you had a colonoscopy?

If you are not exactly sure, please make your best guess. If you have never had a colonoscopy, please leave blank.

Month: 2 Year: 1989

Considering all types of alcoholic beverages, how many times during the past month did you have 4 or more drinks on an occasion?

(If you would rather not answer, please leave the answer blank.)

Answer: 17

During the past month, how many times have you driven when you've had too much to drink?

(If you would rather not answer, please leave the answer blank.)

Answer: 5

When was the last time you had your cholesterol checked? Learn More

If you are not exactly sure, please make your best guess. If you have never had your cholesterol checked, please leave blank. **Month:** 3 **Year:** --

Submit and Continue

(Please just click *once*. The system may take a few moments to respond to your request.)

Any missing or incomplete answers will be marked with RED above.
