# Supplementary material for: Effects of a Web-Based Patient Activation Intervention to Overcome Clinical Inertia on Blood Pressure Control: Cluster Randomized Controlled Trial
Source: J Med Internet Res. 2013 Sep 4;15(9):e158. doi: 10.2196/jmir.2298 (PMC3785979; doi:10.2196/jmir.2298)
Supplement: Supplementary file 8 [file jmir_v15i9e158_app8.pdf]

PREVENTIVE FEEDBACK

Thanks again for your help in this important study! Your job in this study is to speak up and ask questions during your doctor visits. We will NOT be sending any of this information to your doctor or be contacting you with further suggestions for your care. It is up to YOU to take charge of these issues.

You may want to ask your doctor:
WHAT CAN YOU DO TO HELP ME QUIT SMOKING?

From what you've told us, you smoke cigarettes. Many adults who smoke can benefit from talking with a health professional about their smoking. A doctor can work with you to find the best medication or program to help you quit.

Click for more information

You may want to ask your doctor:
WOULD I BENEFIT FROM HAVING A MAMMOGRAM?

You reported that you have not had a mammogram in the past two years. You may want to ask your doctor about this. Many women older than 40 can benefit from having a mammogram every 1-2 years to help detect breast cancer at an early stage.

Click for more information

You may want to ask your doctor:
WOULD I BENEFIT FROM A COLON CANCER SCREENING TEST?

From what you've told us, you haven't had a colon cancer screening test recently. You may want to ask your doctor about this. Many adults age 50 and over can benefit from being screened regularly to help detect colon cancer. There are several tests that can be done to screen for colon cancer. The most common tests are sigmoidoscopy and barium enema (at least every 5 years), colonoscopy (at least every 10 years), and a special kit used at home to determine whether the stool contains blood (at least every year).

Click for more information

You may want to ask your doctor:
WOULD I BENEFIT FROM HAVING A PAP TEST?

From what you've told us, you haven't had a PAP test recently. You may want to ask your doctor about this. Many women 18 and older can benefit from having a PAP test at least every 3 years to detect cervical cancer at an early stage.

Click for more information

You may want to ask your doctor:
WOULD I BENEFIT FROM HAVING A TETANUS VACCINE?

From what you've told us, it's been more than 10 years since your last Tetanus vaccination. You should talk to your doctor about this. Many adults can benefit from having a tetanus vaccine at least every 10 years. Tetanus can cause a deadly infection, so preventing it with a vaccination is essential.

Click for more information

You may want to ask your doctor:
IS MY DRINKING A PROBLEM?

From what you've told us, at least once in the past month you've either had more than a few drinks on one occasion OR you've driven under the influence of alcohol. You may want to ask your doctor about this. Many adults with this pattern of drinking can benefit from talking with a health professional about their alcohol use.

Click for more information

You may want to ask you doctor:
WOULD I BENEFIT FROM HAVING MY CHOLESTEROL CHECKED?

From what you've told us, you haven't had your cholesterol checked recently. You should have this checked when you see your doctor. You should have your cholesterol checked at least every five years. Controlling your cholesterol can lower your risk of a heart attack.

Click for more information

IT'S GREAT THAT YOU'RE TAKING ASPIRIN REGULARLY.

From what you've told us, you either have coronary heart disease or you are at high risk of developing it. Taking 75 – 81 milligrams of aspirin regularly can lower this risk. Aspirin can be dangerous for some people to take. It's important to make sure that your doctor is aware that you take aspirin.

Click for more information

IT'S GREAT THAT YOU'VE HAD A PNEUMONIA VACCINE.

Many adults who are at risk of getting very sick from pneumonia caused by "pneumococcus" can benefit from having a pneumonia vaccine at least one time. People at high risk include anyone over 65 and anyone who has diabetes, lung, or heart problems.

Click for more information

PREVENTIVE FEEDBACK

myexpertdoctor®

ASK THE RIGHT QUESTIONS
GET BETTER CARE

- Questions to Ask Your Doctor
- My Journal
- Resources
- Tips for Talking with Your Doctor
- Contact Us
- Logout

My Journal

Feel free to use this space as you would like. Many people find it helpful to make some notes about what they did on a particular day, and how they felt.

Note: The information you provide in this section is not monitored or reviewed. Information written here will not be shared with any other party - it is for your personal use only, and *Web Quality* assumes no responsibility for any information you choose to enter in your journal. Please note that your ability to access this online journal will terminate once you have completed the study.

Save

My Previous Journal Entries:

myexpertdoctor®

ASK THE RIGHT QUESTIONS
GET BETTER CARE

- Questions to Ask Your Doctor
- My Journal
- Resources
- Tips for Talking with Your Doctor
- Contact Us
- Logout

Resources

Immunizations

Click here to find out more about how vaccines work.

Click here to take a quiz to find out which vaccinations can help you.

Cancer Screening

Click here to learn more about why cancer screening is so important.

Click here to see cancer statistics.

Click here to find out how you can protect yourself against cancer.

Other Screening

Click here to learn more about vision screening.

Click here to learn more about hearing screening.

Click here to learn more about screening for and the prevention of sexually transmitted diseases (STD).

Lifestyle

Long-term Effects Of Smoking

Find out general information on smoking and its effects on the body:
Questions about Smoking, Tobacco, and Health

Learn more about how smoking affects your bones and muscles:
Smoking and Musculoskeletal Health

Learn more about how smoking affects women's ability to get pregnant and carry to term, as well how it affects the children of pregnant smokers before and after birth.
Tobacco Use and Reproductive Outcomes

Find out how smoking affects your eyes.
Smoking and Eye Disease

PREVENTIVE FEEDBACK

myexpertdoctor®

ASK THE RIGHT QUESTIONS
GET BETTER CARE

Lifestyle

Long-term Effects Of Smoking

Find out general information on smoking and its effects on the body:
Questions about Smoking, Tobacco, and Health

Learn more about how smoking affects your bones and muscles:
Smoking and Musculoskeletal Health

Learn more about how smoking affects women's ability to get pregnant and carry to term, as well how it affects the children of pregnant smokers before and after birth.
Tobacco Use and Reproductive Outcomes

Find out how smoking affects your eyes.
Smoking and Eye Disease

Learn more about how smoking affects your skin.
Smoking: How Does It Affect Your Skin?

Learn more about how smoking can affect your digestive system.
Smoking and Your Digestive System

Health Check Tools

Click here to find out your risk of breast cancer.

Click here to find out your risk of colon cancer.

Click here to find out your risk of lung cancer.

Click here to find out your risk of ovarian cancer.

Click here to find out your risk of prostate cancer.

Did You Know....

EPA has concluded that exposure to secondhand smoke can cause lung cancer in adults who do not smoke. EPA estimates that exposure to secondhand smoke causes approximately 3,000 lung cancer deaths per year in nonsmokers.
- Source: Environmental Protection Agency

myexpertdoctor®

ASK THE RIGHT QUESTIONS
GET BETTER CARE

Questions to Ask Your Doctor
My Journal
Resources
Tips for Talking with Your Doctor
Contact Us
Logout

Tips For Talking with Your Doctor

The single most important way you can stay healthy is to be an active member of your own health care team. One way to get high-quality health care is to find and use information and take an active role in all of the decisions made about your care.

This information will help you when talking with your doctor.

Research has shown that patients who have good relationships with their doctors tend to be more satisfied with their care—and tend to have better results. Here are some tips to help you and your doctor become partners in improving your health care.

GIVE INFORMATION. DON'T WAIT TO BE ASKED!

- You know important things about your symptoms and your health history. Tell your doctor what you think he or she needs to know.
- It is important to tell your doctor personal information—even if it makes you feel embarrassed or uncomfortable.
- Bring a "health history" list with you, and keep it up to date. A "health history" is a list that includes any illnesses, diagnoses, tests, and treatments that you've had and any medications that you've taken. You might want to make a copy of the form for each member of your family.
- Always bring any medicines you are taking or a list of those medicines (include when and how often you take them) and what strength. Talk about any allergies or reactions you have had to your medicines.
- Tell your doctor about any herbal products you use or alternative medicines or treatments you receive.
- Bring other medical information, such as x-ray films, test results, and medical records.

GET INFORMATION

- Ask questions. If you don't, your doctor may think you understand everything that was said.
- Write down your questions before your visit. List the most important ones first to make sure they get asked and answered.
- You might want to bring someone along to help you ask questions. This person can also

PREVENTIVE FEEDBACK

myexpertdoctor®

ASK THE RIGHT QUESTIONS
GET BETTER CARE

GET INFORMATION

- Ask questions. If you don't, your doctor may think you understand everything that was said.
- Write down your questions before your visit. List the most important ones first to make sure they get asked and answered.
- You might want to bring someone along to help you ask questions. This person can also help you understand and/or remember the answers.
- Ask your doctor to draw pictures if that might help to explain something.

WHAT IF MY DOCTOR SAYS "NO" TO WHAT I ASK?

- Be patient and listen. Remember that your doctor knows quite a bit about you and may have a good reason for not going along with your plan.
- Ask your doctor "why not"? Your doctor may have a very good point, something that you may not have considered.
- Ask your doctor what might happen that would change his or her mind? Doctors often don't discuss a long-term plan, though they may know it. Your doctor may just be waiting a little while before making the change you asked for.
- Be persistent. Doctors tend to be less aggressive in treating chronic illnesses, such as high blood pressure. You may need to ask again at a later time.

TAKE NOTES

- Some doctors do not mind if you bring a tape recorder to help you remember things. But always ask first.
- Let your doctor know if you need more time. If there is not time that day, perhaps you can speak to a nurse or physician assistant on staff. Or, ask if you can call later to speak with someone.

TAKE INFORMATION HOME

- Ask for written instructions.
- Your doctor also may have brochures, audio tapes, and videotapes that can help you. If not, ask how you can get such materials.

ONCE YOU LEAVE THE DOCTOR'S OFFICE, FOLLOW UP

myexpertdoctor®

ASK THE RIGHT QUESTIONS
GET BETTER CARE

- Be patient and listen. Remember that your doctor knows quite a bit about you and may have a good reason for not going along with your plan.
- Ask your doctor "why not"? Your doctor may have a very good point, something that you may not have considered.
- Ask your doctor what might happen that would change his or her mind? Doctors often don't discuss a long-term plan, though they may know it. Your doctor may just be waiting a little while before making the change you asked for.
- Be persistent. Doctors tend to be less aggressive in treating chronic illnesses, such as high blood pressure. You may need to ask again at a later time.

Questions to Ask Your Doctor

My Journal

Resources

Tips for Talking with Your Doctor

Contact Us

Logout

TAKE NOTES

- Some doctors do not mind if you bring a tape recorder to help you remember things. But always ask first.
- Let your doctor know if you need more time. If there is not time that day, perhaps you can speak to a nurse or physician assistant on staff. Or, ask if you can call later to speak with someone.

TAKE INFORMATION HOME

- Ask for written instructions.
- Your doctor also may have brochures, audio tapes, and videotapes that can help you. If not, ask how you can get such materials.

ONCE YOU LEAVE THE DOCTOR'S OFFICE, FOLLOW UP

- If you have questions, call.
- If your symptoms get worse, or if you have problems with your medicine, call.
- If you had tests and do not hear from your doctor, call for your test results.
- If your doctor said you need to have certain tests, make appointments at the lab or other offices to get them done.
- If your doctor said you should see a specialist, make an appointment.

Remember, quality matters, especially when it comes to your health.

For more on health care quality and materials to help you make health care decisions, go to the "Be an Active Health Care Consumer Pathfinder" at: www.ahrq.gov/path/beactive.htm.
