# Supplementary material for: Effects of a Web-Based Patient Activation Intervention to Overcome Clinical Inertia on Blood Pressure Control: Cluster Randomized Controlled Trial
Source: J Med Internet Res. 2013 Sep 4;15(9):e158. doi: 10.2196/jmir.2298 (PMC3785979; doi:10.2196/jmir.2298)
Supplement: Supplementary file 9 [file jmir_v15i9e158_app9.pdf]

CONSORT-EHEALTH Checklist V1.6.2 Report

(based on CONSORT-EHEALTH V1.6), available at [http://tinyurl.com/consort-ehealth-v1-6].

2298

Date completed

8/7/2012 16:27:40

by

Christopher Sciamanna

Effects of a web-based patient activation intervention to overcome clinical inertia on blood pressure control: a Randomized Controlled Trial

TITLE

1a-i) Identify the mode of delivery in the title

"web-based"

1a-ii) Non-web-based components or important co-interventions in title

This item not relevant to the study as non-web-based components were minimal

1a-iii) Primary condition or target group in the title

"clinical inertia on blood pressure"

ABSTRACT

1b-i) Key features/functionalities/components of the intervention and comparator in the METHODS section of the ABSTRACT

"(1) Intervention Condition – patients used a fully-automated website each month to receive tailored messages that suggested questions to ask their PCP to improve blood pressure control; (2) Control Condition – a similar tool that suggested questions to ask about preventive services (e.g., cancer screening)."

1b-ii) Level of human involvement in the METHODS section of the ABSTRACT

"patients used a fully-automated web-based tool"

1b-iii) Open vs. closed, web-based (self-assessment) vs. face-to-face assessments in the METHODS section of the ABSTRACT

"Primary care providers and their patients with hypertension were recruited by letter"

1b-iv) RESULTS section in abstract must contain use data

"Of 500 enrolled patients (282 intervention and 218 control), 415 (83.0%) completed the 1-year follow up visit. At baseline, 294 (62.3%) of subjects had controlled blood pressure. 411 (82.2%) participants utilized the intervention during at least 6 of 12 months and 222 (62.5%) reported asking questions directly from the web-based tool. There were no group differences, however, in asking about medication intensification. And, after 12 months, there were no differences in blood pressure control between the intervention and control conditions (172 [72.6%] v. 121 [68.0%]; p=.31). More intervention subjects discussed having a creatinine (92 [52.6%] v. 49 [35.5%]; p=.003) and urine protein (81 [44.8%] v. 21 [14.6%]; p <.001) test, but no group differences were observed in the rate of testing, based on chart reviews at 12 months. Control subjects reported more frequent discussions about tetanus and pneumonia vaccines and, after 12 months, reported more frequently receiving both tetanus (30 [13.8%] v. 15 [5.3%]; p= .001) and pneumonia (25 [11.5%] v. 16 [5.7%]; p=.02) vaccination."

1b-v) CONCLUSIONS/DISCUSSION in abstract for negative trials

"The use of a patient activation intervention designed to overcome clinical inertia for hypertension care did not lead to improvements in blood pressure control or greater use of blood pressure medications. Participant adherence to the intervention was high;" "participants were likely to discuss the questions with PCPs. These discussions did not, however, lead to improvements in blood pressure control."

INTRODUCTION

2a-i) Problem and the type of system/solution

"The purpose of this RCT (randomized controlled trial) was to determine whether web-based tailored messages that prompted patients to ask specific questions during visits would improve blood pressure control. The intervention was designed to help participants overcome clinical inertia in their hypertension care."

2a-ii) Scientific background, rationale: What is known about the (type of) system

"Engaging patients in their own care, also known as patient activation, has been increasingly described as a strategy to improve self-management of chronic diseases such as hypertension. Positive changes in measures of patient activation have been associated with improved patient self-management activities among patients with chronic conditions [5]. One important way for patients to be involved in their care is to ask questions during physician visits. Kravitz and colleagues observed that standardized patients who asked for a treatment for depression were more than twice as likely to receive a prescription for an antidepressant medication as standardized patients who made no request [6]. This is consistent with many studies that report that prompting patients to ask their providers specific questions leads to changes in care [7-9]. Reminders, given to patients, about questions they should ask at the point of care have been widely successful in improving preventive care [7,10,11]. What has not been shown, however, is whether these reminders have an impact on the control of chronic illnesses."

METHODS

3a) CONSORT: Description of trial design (such as parallel, factorial) including allocation ratio

"By activating participants to become involved in their own care, we hypothesized that the intervention would increase medication intensification among patients whose blood pressure was not at target, which would thereby increase the percentage of patients who achieved standard blood pressure goals after one year"

3b) CONSORT: Important changes to methods after trial commencement (such as eligibility criteria), with reasons

This item not relevant to the study as there were no changes made and no unexpected events occurred

3b-i) Bug fixes, Downtimes, Content Changes

4a) CONSORT: Eligibility criteria for participants

Exclusion criteria included "Patient did not have personal access to the Internet at home or at work" and "Patient did not have a personal email account"

4a-i) Computer / Internet literacy

4a-ii) Open vs. closed, web-based vs. face-to-face assessments:

"Primary care providers whose practices were located within 40 miles of PSHMC (Penn State Hershey Medical Center) in Central Pennsylvania were recruited. To maximize recruitment of minority patients, online census data were used to create a list of zip codes within 40 miles of PSHMC with the highest racial and ethnic minority populations. A list of PCPs within these zip codes was then purchased from a marketing firm (SK&A, Inc.). Recruitment letters were mailed to providers;" "After consenting the PCP, study staff visited the practice to review the charts of patients to identify eligible patients who met the blood pressure and age criteria (Table 1). Patients meeting these criteria were mailed recruitment letters co-signed by their PCP and the study investigator (CNS). Patients interested in participating were then encouraged to call the toll-free study number;" "Eligible participants were scheduled for a baseline visit at their physician's office, where their blood pressure was measured and their current medications were recorded by study staff. After the baseline visit, subjects were considered enrolled and were assigned to the same condition as their PCP. After the baseline visit, while at home, subjects completed the baseline measures (e.g., demographics) on the study website. During the study, scheduled dates of all visits with their PCP were tracked. After the first visit with their PCP, participants completed an exit survey online to assess that care that was provided during the visit (e.g., topics discussed, medication changes made). At the end of the 12 month study, participants completed follow-up self-reported measures on the study website and met once again in the office of their PCP with a study staff member to measure their blood pressure and record their current medications."

4a-iii) Information giving during recruitment

"Study staff members made follow-up phone calls to assess the level of interest of the physicians in having their practice participate in the study. Project staff visited physicians who expressed interest in order to more fully explain the study and to recruit them into the study." "During a screening phone call, the study was explained to the patient and the patient was assessed for the remaining inclusion and exclusion criteria (Table 1). Eligible participants were scheduled for a baseline visit at their physician's office, where study staff received their consent (see Multimedia Appendix 2)"

Note that Multimedia Appendix 2 is the consent form signed by participants

4b) CONSORT: Settings and locations where the data were collected

"while at home, subjects completed the baseline measures (e.g., demographics) on the study website. During the study, scheduled dates of all visits with their PCP were tracked. After the first visit with their PCP, participants completed an exit survey online to assess that care that was provided during the visit (e.g., topics discussed, medication changes made). At the end of the 12 month study, participants completed follow-up self-reported measures on the study website;" "An important requirement of the intervention was that patients enter data (e.g., blood pressure, creatinine values) that would generally be obtained from office visits. For that reason, we created a wallet-sized "Pocket Chart" to help patients gather this data during office visits. Subject could then later enter these numbers into the website. Patients were encouraged to print the "Pocket Chart" and bring it to their doctor visits and ask their physician to record test results, or ask their physician for the test value and record it themselves."

4b-i) Report if outcomes were (self-)assessed through online questionnaires

"while at home, subjects completed the baseline measures (e.g., demographics) on the study website. During the study, scheduled dates of all visits with their PCP were tracked. After the first visit with their PCP, participants completed an exit survey online to assess that care that was provided during the visit (e.g., topics discussed, medication changes made). At the end of the 12 month study, participants completed follow-up self-reported measures on the study website;" "An important requirement of the intervention was that patients enter data (e.g., blood pressure, creatinine values) that would generally be obtained from office visits. For that reason, we created a wallet-sized "Pocket Chart" to help patients gather this data during office visits. Subject could then later enter these numbers into the website. Patients were encouraged to print the "Pocket Chart" and bring it to their doctor visits and ask their physician to record test results, or ask their physician for the test value and record it themselves."

4b-ii) Report how institutional affiliations are displayed

This item not relevant to the paper as no university affiliation was present on the web-based tool or printable "Pocket Chart"

5) CONSORT: Describe the interventions for each group with sufficient details to allow replication, including how and when they were actually administered

5-i) Mention names, credential, affiliations of the developers, sponsors, and owners

"The authors are distinct from the intervention developers; however, the user interface development was done by Digital Alternatives under contract by authors"

5-ii) Describe the history/development process

"A complete description of the study design and baseline characteristics of participants is published elsewhere"

5-iii) Revisions and updating

This item not relevant to the paper since the intervention did not undergo any major changes during the intervention process.

5-iv) Quality assurance methods

"Blood pressure was measured by a standardized protocol [16]. Based on the blood pressure measurement at the final visit, and the participants' history of diabetes or chronic kidney disease from chart reviews, blood pressure was categorized as controlled or not controlled. Hypertension guidelines from JNC 7 suggested that patients with diabetes or chronic kidney disease should have a blood pressure target of less than 130/80 and all other adults should have a blood pressure target of less than 140/90;" "Changes in medications as well as hypertension-related tests (e.g., creatinine) were measured by chart abstraction at 12 months. The use of preventive services (e.g., Tetanus vaccination) was measured via patient self-report. The impact of the intervention on doctor-patient communication was measured by a self-reported survey, completed within 72 hours after the subjects' visit with their hypertension care provider. This exit survey was designed to measure what was discussed during the visit, and provide insight into how the tailored feedback was being used. Similar methods have been studied by one of the investigators (CNS) and observed to be accurate for identifying activities performed during provider visits [17]."

5-v) Ensure replicability by publishing the source code, and/or providing screenshots/screen-capture video, and/or providing flowcharts of the algorithms used

Screenshots are provided for the monthly surveys and feedback

5-vi) Digital preservation

This item not relevant to the paper as the website has not been archived and can no longer be accessed.

5-vii) Access

"After the baseline visit, subjects completed the baseline measures (e.g., demographics) on the study website using their personal work or home computer;" "After the first visit with their PCP, participants completed an exit survey online to assess that care that was provided during the visit (e.g., topics discussed, medication changes made). At the end of the 12 month study, participants completed follow-up self-reported measures on the study website;" "There was no cost associated with using the study website"

5-viii) Mode of delivery, features/functionalities/components of the intervention and comparator, and the theoretical framework

“The patient was then provided with on-screen tailored feedback, based on pre-programmed rules adapted from recommendations in JNC 7 [2]. For any situation in which the patient appeared to be in need of a change to their care (e.g., BP was higher than JNC 7 goals), the tailored feedback also included a question that the participant should consider asking at the next visit (e.g., “Can I lower my blood pressure by drinking less alcohol?”). Participants also receive a layperson description of the scientific rationale for the statement, and a link to a reputable external website that validates asking the suggested question (e.g., American Heart Association). The feedback was ordered so that the highest priority recommendations appeared closest to the top of the page.

The web-based feedback was based on the most recent hypertension guidelines in JNC 7 [2]. JNC 7 was reviewed for the presence of specific recommendations on hypertension management. Recommendations were ranked based on the strength of the evidence supporting them in addition to the likelihood of impact. These recommendations were reviewed by the study’s clinical hypertension expert”

5-ix) Describe use parameters

“participants were instructed to answer questions online once each month and before any visits with their hypertension care provider;” “The intervention was designed to be used before a visit with the physician who provided the patient’s hypertension care. For that reason, it was essential to track the dates of these visits so the patients could be reminded to use the intervention before these visits. We assumed that the intervention would be significantly less effective if used long before or following an office visit, as the intervention is designed to “activate” patients to ask specific questions during visits.”

5-x) Clarify the level of human involvement

“Eligible participants were scheduled for a baseline visit at their physician’s office, where their blood pressure was measured and their current medications were recorded by study staff;” “At the end of the 12 month study, participants completed follow-up self-reported measures on the study website and met once again in the office of their PCP with a study staff member to measure their blood pressure and record their current medications;” “Recommendations were ranked based on the strength of the evidence supporting them in addition to the likelihood of impact. These recommendations were ranked based on the strength of the evidence supporting them in addition to the likelihood of impact. These recommendations were reviewed by the study’s clinical hypertension expert”

5-xi) Report any prompts/reminders used

“participants in both conditions received monthly email reminders to use the intervention, in large part to clarify the date of the next hypertension care provider visit. In addition, patients in both conditions received email reminders to use the site, starting 10 days before their physician visit and repeated twice if the participant has not used the site before the planned visit.”

5-xii) Describe any co-interventions (incl. training/support)

This item not relevant to the paper as there were no co-interventions

6a) CONSORT: Completely defined pre-specified primary and secondary outcome measures, including how and when they were assessed

“The use of preventive services (e.g., Tetanus vaccination) was measured via patient self-report. The impact of the intervention on doctor-patient communication was measured by a self-reported survey, completed within 72 hours after the subjects’ visit with their hypertension care provider. This exit survey was designed to measure what was discussed during the visit, and provide insight into how the tailored feedback was being used. Similar methods have been studied by one of the investigators (CNS) and observed to be accurate for identifying activities performed during provider visits [17].”

6a-i) Online questionnaires: describe if they were validated for online use and apply CHERRIES items to describe how the questionnaires were designed/deployed

6a-ii) Describe whether and how “use” (including intensity of use/dosage) was defined/measured/monitored

“After the first visit with their PCP, participants completed an exit survey online to assess that care that was provided during the visit (e.g., topics discussed, medication changes made). At the end of the 12 month study, participants completed follow-up self-reported measures on the study website;” “Participants were expected to use the website at least once each month”

The following text appears in the Results section where fidelity is discussed: “Adherence was monitored electronically by number of months in which participants logged in.”

6a-iii) Describe whether, how, and when qualitative feedback from participants was obtained

This item not relevant to the paper as there was no qualitative feedback obtained from participants during this study. However, qualitative feedback was obtained in previously published web-based studies on asthma and osteoarthritis done by the authors and considered during the development of this intervention.

6b) CONSORT: Any changes to trial outcomes after the trial commenced, with reasons

This item not relevant to the paper as there were no changes to trial outcomes

7a) CONSORT: How sample size was determined

7a-i) Describe whether and how expected attrition was taken into account when calculating the sample size

“A complete description of the study design and baseline characteristics of participants is published elsewhere”

7b) CONSORT: When applicable, explanation of any interim analyses and stopping guidelines

This item not relevant to the paper as there were no interim analyses.

8a) CONSORT: Method used to generate the random allocation sequence

“Randomization was done at the level of the PCP. PCPs were enrolled and randomized into one of two conditions by selecting an envelope containing a document noting the assigned condition (Intervention or Control) from a stack of sealed envelopes. All patient participants were assigned to the same condition as their PCP. This was done to eliminate contamination, as the intervention had the potential to change the care that the PCP may provide to other patients in the practice.”

8b) CONSORT: Type of randomisation; details of any restriction (such as blocking and block size)

“Randomization was done at the level of the PCP. PCPs were enrolled and randomized into one of two conditions (Intervention or Control). All patient participants were assigned to the same condition as their PCP.”

9) CONSORT: Mechanism used to implement the random allocation sequence (such as sequentially numbered containers), describing any steps taken to conceal the sequence until interventions were assigned

"A complete description of the study design and baseline characteristics of participants is published elsewhere"

10) CONSORT: Who generated the random allocation sequence, who enrolled participants, and who assigned participants to interventions

“Project staff visited physicians who expressed interest in order to more fully explain the study and to recruit them into the study;” “During a screening phone call, the study was explained to the patient and the patient was assessed for the remaining inclusion and exclusion criteria”

11a) CONSORT: Blinding - If done, who was blinded after assignment to interventions (for example, participants, care providers, those assessing outcomes) and how

11a-i) Specify who was blinded, and who wasn’t

“The study was designed as a double-blinded randomized controlled trial; neither study staff nor care providers knew the participants’ group”

11a-ii) Discuss e.g., whether participants knew which intervention was the “intervention of interest” and which one was the “comparator”

“All patient participants were assigned to the same condition as their PCP. This was done to eliminate contamination, as the intervention had the potential to change the care that the PCP may provide to other patients in the practice.”

11b) CONSORT: If relevant, description of the similarity of interventions

“The CC (control condition) was identical to the IC, except that the content of the CC intervention was focused on preventive services, rather than hypertension. The content of the questions and feedback on the website focused on non- hypertension-related preventive services (e.g., mammography screening, tetanus immunizations) recommended by the USPSTF. The CC participants received the same components of the intervention as IC subjects (e.g., web-based personalized feedback, pocket chart and automated email reminders), but the content area focus on preventive services, rather than hypertension. The control condition, being an active treatment control condition, was designed to improve preventive care and not hypertension care. For example, it was designed not to provide feedback about increasing physical activity, which can lower blood pressure. This control condition design was chosen to limit attrition and control for contact time.”

12a) CONSORT: Statistical methods used to compare groups for primary and secondary outcomes

This item not relevant to the paper given high fidelity. The imputation techniques outlined in the data analysis plan were not used.

12a-i) Imputation techniques to deal with attrition / missing values

This item not relevant to the paper given high fidelity. The imputation techniques outlined in the data analysis plan were not used.

12b) CONSORT: Methods for additional analyses, such as subgroup analyses and adjusted analyses

“Subgroup analyses were performed to understand the impact of the intervention on individuals whose blood pressure was uncontrolled at baseline.”

RESULTS

13a) CONSORT: For each group, the numbers of participants who were randomly assigned, received intended treatment, and were analysed for the primary outcome

“Of the physicians contacted, 54 responded and agreed to participate (see Figure 1);” “Eligible participants (n = 528) were scheduled for a baseline visit where three consecutive blood pressures were measured, and they were enrolled in the study. Of those scheduled, 500 completed the baseline visit and 218 subjects enrolled into the CC (prevention) and 282 into the IC (hypertension).”

13b) CONSORT: For each group, losses and exclusions after randomisation, together with reasons

“See Figure 1 for CONSORT diagram of participant flow.”

13b-i) Attrition diagram

14a) CONSORT: Dates defining the periods of recruitment and follow-up

“During our study period, there was an established trend of increasing treatment and control of blood pressure. This national trend is as of 2008; more recent national data is not available.”

14a-i) Indicate if critical “secular events” fell into the study period

14b) CONSORT: Why the trial ended or was stopped (early)

This item not relevant to the paper as the trial did not end early

15) CONSORT: A table showing baseline demographic and clinical characteristics for each group

“Table 2 reports the baseline characteristics. There were no significant differences in most variables between study groups, including the demographic variables of age, gender, race, ethnicity, education, and income.”

15-i) Report demographics associated with digital divide issues

“Table 2 reports the baseline characteristics. There were no significant differences in most variables between study groups, including the demographic variables of age, gender, race, ethnicity, education, and income.”

16a) CONSORT: For each group, number of participants (denominator) included in each analysis and whether the analysis was by original assigned groups

16-i) Report multiple “denominators” and provide definitions

“500 completed the baseline visit and 218 subjects enrolled into the CC (prevention) and 282 into the IC (hypertension). Following the baseline visit, the majority (95.2%) of participants (n = 476) logged onto the website and completed the Online Baseline Measures. From the 476 participants who completed the baseline measures questionnaire, demographic data as well as baseline secondary outcome data were collected (Table 2). Following their first visit with their PCP, 363 participants completed a survey to record what occurred during the visit. After 12 months, 83.6% (n= 418) returned for their follow up visit”

16-ii) Primary analysis should be intent-to-treat

“Data analysis was focused on the primary hypothesis that a higher percentage of participants in the IC condition, compared to CC participants, would have controlled blood pressure at 12 months, using intent-to-treat principles [21]. This was done using Pearson’s chi-squared test. The effects of the intervention on continuous blood pressure values were then compared using the Student’s t-test. Logistic regression was performed to understand the impact of variables that differed between conditions at baseline (number of BP medications and employment status).”

17a) CONSORT: For each primary and secondary outcome, results for each group, and the estimated effect size and its precision (such as 95% confidence interval)

“Data analysis was focused on the primary hypothesis that a higher percentage of participants in the IC condition, compared to CC participants, would have controlled blood pressure at 12 months, using intent-to-treat principles [21]. Overall rate of blood pressure control was the primary outcome as it was powered to detect differences in participants with controlled blood pressure. This was done using Pearson’s chi-squared test. The effects of the intervention on continuous blood pressure values were then compared using the Student’s t-test. Logistic regression was performed to understand the impact of variables that differed between conditions at baseline (number of BP medications and employment status).”

17a-i) Presentation of process outcomes such as metrics of use and intensity of use

17b) CONSORT: For binary outcomes, presentation of both absolute and relative effect sizes is recommended

“Table 3 reports blood pressure outcomes at 12 months. The overall rate of participants with controlled blood pressure increased from 294 out of 472 (62.3%) at baseline to 293 out of 415 (70.6%) at 12 months. No significant difference was observed between study groups with respect to rates of blood pressure control (172 out of 237 intervention participants [72.6%] v. 121 out of 178 control [68.0%]; p=.31). Similar results were observed when blood pressure was examined as a continuous variable and when the results were expressed as continuous changes within groups. For example, the mean (SD) systolic blood pressure at 12 months was not significantly different between conditions [128.3 (13.4) among IC subjects, 129.4 (14.1) among CC subjects; p=.42];” “222 out of 355 (62.5%) subjects reported asking a question that was suggested by the tailored feedback, during the visit with their PCP. As expected, there were significant differences in the specific topics discussed during visits by IC and CC subjects. More CC subjects than IC subjects, for example, discussed having a tetanus vaccine (50 out of 141 [35.5%] v. 28 out of 166 [16.9%]; p<.001) and a pneumonia vaccination (39 out of 135 [28.9%] v. 23 out of 160 [14.4%]; p=.002). Similarly, more IC subjects than CC subjects discussed having serum creatinine tested (92 out of 175 [52.6%] v. 49 out of 138 [35.5%]; p=.003) and urine protein tested (81 out of 181 [44.8%] v. 21 out of 144 [14.6%]; p<.001).”

18) CONSORT: Results of any other analyses performed, including subgroup analyses and adjusted analyses, distinguishing pre-specified from exploratory

“Since the goal of the intervention was to intervene on patients with uncontrolled blood pressure, a subgroup analysis was performed that was limited to these participants whose blood pressure was uncontrolled at baseline. Of the 152 participants found to be uncontrolled at baseline, 45.4% were controlled at 12-month follow-up. However, no significant difference was observed in blood pressure control rates between study groups”

18-i) Subgroup analysis of comparing only users

19) CONSORT: All important harms or unintended effects in each group

This item not relevant to the paper as there were no privacy breaches, technical problems or other unintended effects.

19-i) Include privacy breaches, technical problems

19-ii) Include qualitative feedback from participants or observations from staff/researchers

DISCUSSION

20) CONSORT: Trial limitations, addressing sources of potential bias, imprecision, multiplicity of analyses

20-i) Typical limitations in ehealth trials

“patients may not have used the intervention before doctor visits. Overall adherence rates were relatively high (Figure 2), however, with most (82.2%) participants using the intervention each month. This does not guarantee, however, that patients came to the visit with the printout of their questions to ask and, in fact, asked one or more of the questions that the feedback suggested;” “Second, a limitation of the study was the management and use of blood pressure values. Patients were encouraged to enter the most recent blood pressure value into the website, which then generated tailored feedback based, in part, on that number. Approximately one-third, however, entered no blood pressure value, as they were unaware of their blood pressure. Even if the subject had entered a blood pressure value, during the subsequent visit to their provider that value would likely have been different. This difference may have led to different tailored messages and may, therefore, have created uncertainty for patients, undermining their desire to ask for treatment intensifications.”

21) CONSORT: Generalisability (external validity, applicability) of the trial findings

21-i) Generalizability to other populations

“home BP may be underutilized for informing providers about whether a patients’ blood pressure is controlled. By moving the data from the provider to the patient, patient activation interventions such as this may have a greater chance of improving BP control;” “Despite low rates of adherence to web-based interventions [29,30], we observed high rates of fidelity (>70% of all study months) by asking patients to complete a brief web-based survey, leading to tailored feedback, each month and before provider visits. This intervention structure, monthly use of online tool, could be used to impact the care of patients experiencing a range of conditions.”

21-ii) Discuss if there were elements in the RCT that would be different in a routine application setting

“Control condition subjects, however, who were prompted to ask questions about preventive services, were more likely to receive a tetanus vaccine (20.7% v. 8.7%; p=0.01), as well as a pneumonia vaccine (11.5% v. 5.7%; p= 0.02). These findings were consistent with previous studies observing that patient reminders improve preventive service utilization;” “We purposefully chose not to prompt the doctor separately from the patient, as the goal of the study was to examine patient activation, not provider activation.”

22) CONSORT: Interpretation consistent with results, balancing benefits and harms, and considering other relevant evidence

22-i) Restate study questions and summarize the answers suggested by the data, starting with primary outcomes and process outcomes (use)

“The overall rate of participants with controlled blood pressure increased from 311.5 out of 500 (62.3%) at baseline to 293 out of 415 (70.6%) at 12 months. No significant difference was observed between study groups with respect to rates of blood pressure control (72.6% intervention, 68.0% control; p=0.31);” “More than half (62.5%) of subjects reported asking a question that was suggested by the tailored feedback, during the visit with their PCP. As expected, there were significant differences in the specific topics discussed during visits by IC and CC subjects. More CC subjects than IC subjects, for example, discussed having a tetanus vaccine (35.5% v. 16.9%; p<0.01) and a pneumonia vaccination (28.9% v. 14.4%; p<0.01). Similarly, more IC subjects than CC subjects discussed having serum creatinine tested (52.6% v. 35.5%; p<0.01) and urine protein tested (44.8% v. 14.6%; p<0. 01).”

22-ii) Highlight unanswered new questions, suggest future research

The following text occurs in the Discussion section where resulting questions and suggestions are discussed: “It is possible that, if home monitoring had been used, that patients may have been more likely to ask for medication intensifications. This situation would be more akin to depression, where the patient has all of the data, which doctors typically do not ask for. We hypothesize that if a patient entered the visit, as suggested by some investigators [23], with an average of 10 blood pressure values, which he/she knew were too high, the results of the study may have been different;” “While patients with symptomatic conditions (e.g., asthma, migraines) are prompted to ask for treatment intensifications in order to feel better, patients with conditions that have few symptoms (e.g., hypertension, high cholesterol, type 2 diabetes) lack the symptom trigger to request treatment intensifications.”

Other information

23) CONSORT: Registration number and name of trial registry

“ClinicalTrials.gov NCT00377208”

24) CONSORT: Where the full trial protocol can be accessed, if available

“http://www.clinicaltrials.gov/ct2/show/NCT00377208?term=web-based+blood+pressure&rank=1”

25) CONSORT: Sources of funding and other support (such as supply of drugs), role of funders

“This study was funded by the National Heart, Lung and Blood Institute, grant# R01HL083432”

X26-i) Comment on ethics committee approval

“The protocol and all consent forms were approved by the Institutional Review Board of the Pennsylvania State University College of Medicine.”

x26-ii) Outline informed consent procedures

“Project staff visited physicians who expressed interest in order to more fully explain the study and to recruit them into the study.

After consenting the PCP, study staff visited the practice to review the charts of patients to identify eligible patients who met the blood pressure and age criteria (Table 1). Patients meeting these criteria were mailed recruitment letters co-signed by their PCP and the study investigator (see Multimedia Appendix 1). Patients interested in participating were then encouraged to call the toll-free study number. During a screening phone call, the study was explained to the patient and the patient was assessed for the remaining inclusion and exclusion criteria (Table 1).

Eligible participants were scheduled for a baseline visit at their physician’s office, Eligible participants were scheduled for a baseline visit at their physician’s office, where study staff received their consent (see Multimedia Appendix 2). After the baseline visit, subjects were considered enrolled and were assigned to the same condition as their PCP.”

Multimedia Appendix 2 is the consent form signed by participants

X26-iii) Safety and security procedures

“Blood pressure was measured by a standardized protocol;” “participants completed follow-up self-reported measures on the study website (see Multimedia Appendix 4); these measures includes questions regarding adverse events that occurred during their participation in the study and whether these events were related to the study.”

X27-i) State the relation of the study team towards the system being evaluated

“The authors have no conflicts of interest. The authors are distinct from the intervention developers; however, the user interface development was done by Digital Alternatives under contract by authors.”
